# Phase Transition in Frustrated Magnetic Thin Film—Physics at Phase Boundaries

**DOI:** 10.3390/e21020175

**Published:** 2019-02-13

**Authors:** Hung T. Diep

**Affiliations:** Laboratoire de Physique Théorique et Modélisation, Université de Cergy-Pontoise, CNRS, UMR 80892, Avenue Adolphe Chauvin, CEDEX, 95302 Cergy-Pontoise, France; diep@u-cergy.fr; Tel.: +33-1-3425-7501

**Keywords:** frustration, phase transition, reentrance, disorder lines, surface spin waves, magnetic thin films, theory, simulation

## Abstract

In this review, we outline some principal theoretical knowledge of the properties of frustrated spin systems and magnetic thin films. The two points we would like to emphasize: (i) the physics in low dimensions where exact solutions can be obtained; (ii) the physics at phase boundaries where interesting phenomena can occur due to competing interactions of the two phases around the boundary. This competition causes a frustration. We will concentrate our attention on magnetic thin films and phenomena occurring near the boundary of two phases of different symmetries. Two-dimensional (2D) systems are in fact the limiting case of thin films with a monolayer. Naturally, we will treat this case at the beginning. We begin by defining the frustration and giving examples of frustrated 2D Ising systems that we can exactly solve by transforming them into vertex models. We will show that these simple systems already contain most of the striking features of frustrated systems such as the high degeneracy of the ground state (GS), many phases in the GS phase diagram in the space of interaction parameters, the reentrance occurring near the boundaries of these phases, the disorder lines in the paramagnetic phase, and the partial disorder coexisting with the order at equilibrium. Thin films are then presented with different aspects: surface elementary excitations (surface spin waves), surface phase transition, and criticality. Several examples are shown and discussed. New results on skyrmions in thin films and superlattices are also displayed. By the examples presented in this review we show that the frustration when combined with the surface effect in low dimensions gives rise to striking phenomena observed in particular near the phase boundaries.

## 1. Introduction

Extensive investigations on materials have been carried out over the past three decades. This is due to an enormous number of industrial applications which drastically change our lifestyle. The progress in experimental techniques, the advance on theoretical understanding, and the development of high-precision simulation methods together with the rapid increase of computer power have made possible the rapid development in material science. Today, it is difficult to predict what will be discovered in this research area in ten years.

The purpose of this review is to look back at early and recent results in the physics of frustrated spin systems at low dimensions: 2D systems and magnetic thin films. We would like to connect these results, published over a large period of time, on a line of thoughts: physics at phase boundaries. A boundary between two phases of different orderings is determined as a compromise between competing interactions, each of which favors one kind of ordering. The frustration is thus minimum on the boundary (see reviews on many aspects of frustrated spin systems in Ref. [1]). When an external parameter varies, this boundary changes and we will see in this review that many interesting phenomena occur in the boundary region. We will concentrate on the search for interesting physics near the phase boundaries in various frustrated spin systems in this review.

In the 1970s, statistical physics with Renormalization Group analysis greatly contributed to the understanding of the phase transition from an ordered phase to a disordered phase [2,3]. We will show methods to study the phase transition in magnetic thin films where surface effects when combined with frustration effects give rise to many new phenomena. Physical properties of solid surfaces, thin films, and superlattices have been intensively studied due to their many applications [4,5,6,7,8,9,10,11].

A large part of this review, Section 2, is devoted to the definition of the frustration and to models which are exactly solved. We begin in Section 3 with exactly solved models to have all properties defined without approximation. As seen, many striking phenomena are exactly uncovered such as partial disorder, reentrance, disorder lines, and multiple phase transitions. Only exact mathematical techniques can allow us to reveal such beautiful phenomena which occur around the boundary separating two phases of different ground-state orderings. These exact results permit to understand similar behaviors in systems that cannot be solved such as 3D systems.

In Section 4, an introduction on surface effects in magnetic thin films is given. To avoid a dispersion of techniques, I introduce only the Green’s function method which can be generalized in more complicated cases such as non-collinear spin states. Calculations of the spin-wave spectrum and the surface magnetization are in particular explained.

In Section 5 several striking results obtained mainly by the author’s group are shown on several frustrated magnetic thin films including helimagnetic films. We show in particular the surface phase transition, quantum fluctuations at low temperature, and the existence of partial phase transition. Results obtained by Monte Carlo simulations are also shown in most cases to compare with the Green’s function technique.

The question of the criticality in thin films is considered in Section 6. Here, the high-precision multi-histogram techniques are used to show that critical exponents in magnetic thin films are effective exponents with values between those of the 2D and 3D universality classes.

Section 7 is devoted to skyrmions, a hot subject at the time being due to their numerous possible applications. Here again, we show only results obtained in the author’s group, but we mention a large bibliography. Skyrmions are topological excitations. Skyrmions are shown to result from the competition of different antagonist interactions under an applied magnetic field. We find the existence of a skyrmion crystal, namely a network of periodically arranged skyrmions. Results show that such a skyrmion crystal is stable up to a finite temperature. The relaxation time of skyrmions is shown to follow a stretched exponential law.

Concluding remarks are given in Section 8.

## 2. Physics in Two Dimensions: Frustration Effects

### 2.1. Frustration

Since the 1980s, frustrated spin systems have been subjects of intensive studies [1]. The word “frustration” has been introduced to describe the fact that a spin cannot find an orientation to *fully* satisfy all interactions with its neighbors, namely the energy of a bond is not the lowest one [12,13]. This will be seen below for Ising spins where at least one among the bond with the neighbors is not satisfied. For vector spins, frustration is shared by all spins so that all bonds are only partially satisfied, i.e., the energy of each bond is not minimum. Frustration results either from the competing interactions or from the lattice geometry such as the triangular lattice with antiferromagnetic nearest-neighbor (nn) interaction, the face-centered cubic (fcc) antiferromagnet and the antiferromagnetic hexagonal-close-packed (hcp) lattice (see [1]).

Note that real magnetic materials have complicated interactions and there are large families of frustrated systems such as the heavy lanthanides metals (holmium, terbium and dysprosium) [14,15], helical MnSi [16], pyrochore antiferromagnets [17], and spin-ice materials [18]. Exact solutions on simpler systems may help understand qualitatively real materials. Besides, exact results can be used to validate approximations.

**Figure 1:**
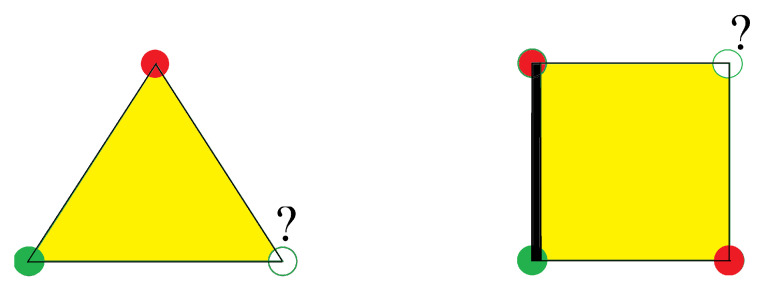
Two frustrated cells are shown. The thin (heavy) lines denote the ferromagnetic (antiferromagnetic) bonds. Up and down spins are shown by green and red circles, respectively. Question marks indicate undetermined spin orientation. Choosing an orientation for the spin marked by the question mark will leave one of its bonds unsatisfied (frustrated bond with positive energy).

We recall in the following some basic arguments leading to the definition of the frustration. The interaction energy of two spins $S_{i}$ and $S_{j}$ interacting with each other by *J* is written as $E=-J\left(S_{i}\cdot S_{j}\right)$. If *J* is ferromagnetic (J>0) then the minimum of *E* is −J corresponding to $S_{i}$ parallel to $S_{j}$. If *J* is antiferromagnetic (J<0), *E* is minimum when $S_{i}$ is antiparallel to $S_{j}$. One sees that in a crystal with nn ferromagnetic interaction, the ground state (GS) of the system is the configuration where all spins are parallel: the interaction of every pair of spins is “fully” satisfied, namely the bond energy is equal to −J. This is true for any lattice structure. If *J* is antiferromagnetic, the GS depends on the lattice structure: (i) for lattices containing no elementary triangles, i.e., bipartite lattices (such as square lattice, simple cubic lattices, …) in the GS each spin is antiparallel to its neighbors, i.e., every bond is fully satisfied, its energy is equal to −|J|; (ii) for lattices containing elementary triangles such as the triangular lattice, the fcc lattice, and the hcp lattice, one cannot construct a GS where all bonds are fully satisfied (see Figure 1). The GS does not correspond to the minimum interaction energy of every spin pair: the system is frustrated.

Let us formally define the frustration. We consider an elementary lattice cell which is a polygon formed by faces called “plaquettes”. For example, the elementary cell of the simple cubic lattice is a cube with six square plaquettes, the elementary cell of the fcc lattice is a tetrahedron formed by four triangular plaquettes. According to the definition of Toulouse [12] the plaquette is frustrated if the parameter *P* defined below is negative

(1) $$P=\prod_{\langle i,j\rangle }\mathrm{sign}\left(J_{i,j}\right),$$

where $J_{i,j}$ is the interaction between two nn spins of the plaquette and the product is performed over all $J_{i,j}$ around the plaquette.

We show two examples of frustrated plaquettes in Figure 1, a triangle with three antiferromagnetic bonds and a square with three ferromagnetic bonds and one antiferromagnetic bond. *P* is negative in both cases. If one tries to put Ising spins on those plaquettes, at least one of the bonds around the plaquette will not be satisfied. For vector spins, we show below that the frustration is equally shared by all bonds so that in the GS, each bond is only partially satisfied.

One sees that for the triangular plaquette, the degeneracy is three, and for the square plaquette it is four. Therefore, the degeneracy of an infinite lattice for these cases is infinite, unlike the non-frustrated case.

The frustrated triangular lattice with nn interacting Ising spins was studied in 1950 [19,20].

### 2.2. Non-Collinear Spin Configurations

For vector spins, non-collinear configurations due to competing interactions were found in 1959 independently by Yoshimori [21], Villain [22] and Kaplan [23].

We emphasize that the frustration may be due to the competition between a Heisenberg exchange model which favors a collinear spin configuration and the Dzyaloshinski-Moriya interaction $E=-D\cdot \left(S_{i}\wedge S_{j}\right)$ [24,25] which favors the perpendicular configuration. We will return to this interaction in the section on skyrmions later in this paper.

We show below how to determine the GS of some frustrated systems and discuss some of their properties.

We consider the plaquettes shown in Figure 1 with XY spins. The GS configuration corresponds to the minimum of the energy *E* of the plaquette. In the case of the triangular plaquette, suppose that spin $S_{i}$(i=1,2,3) of amplitude *S* makes an angle $\theta_{i}$ with the Ox axis. One has

(2) $$\begin{array}{ccc} E & = & -J(S_{1}\cdot S_{2}+S_{2}\cdot S_{3}+S_{3}\cdot S_{1})\\ & = & -JS^{2}\left[\cos\left(\theta_{1}-\theta_{2}\right)+\cos\left(\theta_{2}-\theta_{3}\right)+\cos\left(\theta_{3}-\theta_{1}\right)\right] \end{array}$$

where J<0 (antiferromagnetic). Minimizing *E* with respect to 3 angles $\theta_{i}$, we find the solution $\theta_{1}-\theta_{2}=\theta_{2}-\theta_{3}=\theta_{3}-\theta_{1}=2\pi /3$.

One can also write

$$E=-J\left(S_{1}\cdot S_{2}+S_{2}\cdot S_{3}+S_{3}\cdot S_{1}\right)=+\frac{3}{2}JS^{2}-\frac{J}{2}{\left(S_{1}+S_{2}+S_{3}\right)}^{2}.$$

*J* is negative, the minimum thus corresponds to $S_{1}+S_{2}+S_{3}=0$ which gives the $120^{\circ }$ structure. This is true also for the Heisenberg spins.

**Figure 2:**
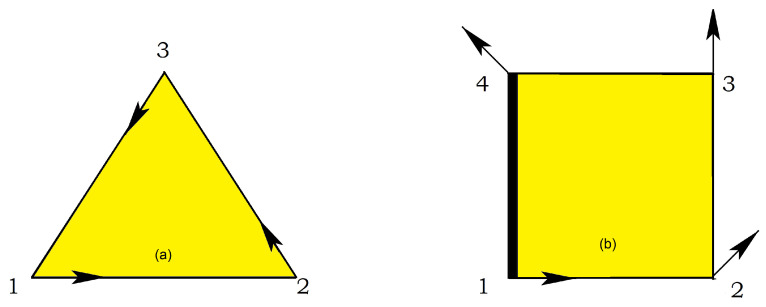
Ground state of XY spins on frustrated triangular and square cells: non-collinear spin arrangements. The thin lines denote the ferromagnetic interaction, the thick line is the antiferromagnetic one.

For the frustrated square plaquette, we suppose that the ferromagnetic bonds are *J* and the antiferromagnetic bond is −J connecting the spins $S_{1}$ and $S_{4}$ (see Figure 2). The energy minimization gives

(3) $$\theta_{2}-\theta_{1}=\theta_{3}-\theta_{2}=\theta_{4}-\theta_{3}=\frac{\pi }{4}\;\mathrm{and}\;\theta_{1}-\theta_{4}=\frac{3\pi }{4}$$

If the antiferromagnetic interaction is −ηJ (η>0), the angles are [26]

(4) $$\cos\theta_{21}=\cos\theta_{32}=\cos\theta_{43}\equiv \theta =\frac{1}{2}{\left[\frac{\eta +1}{\eta }\right]}^{1/2}$$

and $|\theta_{14}\left|=3|\theta \right|$, where $\cos\theta_{ij}\equiv \cos\theta_{i}-\cos\theta_{j}$. This solution exists if |cosθ|≤1, namely $\eta >\eta_{c}=1/3$. One recovers when η=1, θ=π/4, $\theta_{14}=3\pi /4$.

The GS spin configurations of the frustrated triangular and square lattices are displayed in Figure 2 with XY spins. We see that the frustration is shared by all bonds: the energy of each bond is −0.5J for the triangular lattice, and $-\sqrt{2}J/2$ for the square lattice. Thus, the bond energy in both cases is not fully satisfied, namely not equal to −J, as we said above when defining the frustration.

**Figure 3:**
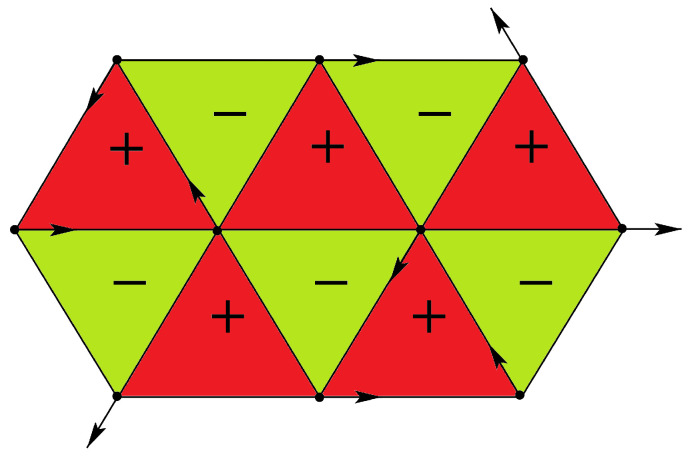
Triangular antiferromagnet with XY spins: the left (right) chirality is indicated by + (−). See text.

At this stage, we note that the GS found above have a two-fold degeneracy resulting from the equivalence of clockwise and counter-clockwise turning angle (noted by + and − in Figure 3) between adjacent spins on a plaquette in Figure 2. Therefore the symmetry of these spin systems is of Ising type O(1), in addition to the symmetry SO(2) due to the invariance by global spin rotation in the plane.

From the GS symmetry, one expects that the respective breaking of O(1) and SO(2) symmetries would behave respectively as the 2D Ising universality class and the Kosterlitz-Thouless transition [3]. However, the question of whether the two phase transitions would occur at the same temperature and the nature of their universality remains an open question [26,27].

Let us determine the GS of a helimagnet. Consider the simplest case: a chain of Heisenberg spins with ferromagnetic interaction $J_{1}\left(>0\right)$ between nn and antiferromagnetic interaction $J_{2}\left(<0\right)$ between nnn. The interaction energy is

(5) $$\begin{array}{ccc} E & = & -J_{1}\sum_{i}S_{i}\cdot S_{i+1}+\left|J_{2}\right|\sum_{i}S_{i}\cdot S_{i+2}\\ & = & S^{2}\left[-J_{1}\cos\theta +\left|J_{2}\right|\cos\left(2\theta \right)\right]\sum_{i}1\\ \frac{\partial E}{\partial \theta } & = & S^{2}\left[J_{1}\sin\theta -2\left|J_{2}\right|\sin\left(2\theta \right)\right]\sum_{i}1=0\\ & = & S^{2}\left[J_{1}\sin\theta -4\left|J_{2}\right|\sin\theta \cos\theta \right]\sum_{i}1=0, \end{array}$$

where one has supposed that the angle between nn spins is θ. The first solution is

sinθ=0⟶θ=0whichistheferromagneticsolution

and the second one is

(6) $$\cos\theta =\frac{J_{1}}{4|J_{2}|}⟶\theta =\pm \arccos\left(\frac{J_{1}}{4|J_{2}|}\right).$$

**Figure 4:**
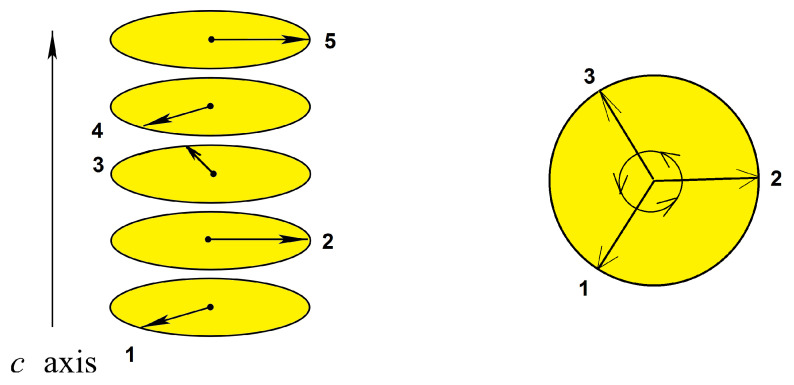
Example of a helimagnetic configuration using $\varepsilon =|J_{2}|/J_{1}>\varepsilon_{c}=1/4$ ($J_{1}>0$, $J_{2}<0$), namely θ=2π/3. Left: 3D view. Right: top view.

This solution is possible when −1≤cosθ≤1, i.e., when $J_{1}/\left(4|J_{2}|\right)\leq 1$ or $|J_{2}|/J_{1}\geq 1/4\equiv \varepsilon_{c}$. An example of configuration is shown in Figure 4. Please note that there are two degenerate configurations of clockwise and counter-clockwise turning angles as other examples above.

Please note that the two frequently studied frustrated spin systems are the fcc and hcp antiferromagnets. These two magnets are constructed by stacking tetrahedra with four frustrated triangular faces. Frustration by the lattice structure such as these cases are called “geometry frustration”. Another 3D popular model which has been extensively studied since 1984 is the system of stacked antiferromagnetic triangular lattices (satl). The phase transition of this system with XY and Heisenberg spins was a controversial subject for more than 20 years. The controversy was ended with our works: the reader is referred to Refs. [28,29] for the history. In short, we found that in known 3D frustrated spin systems (fcc, hcp, satl, helimagnets, …) with Ising, XY, or Heisenberg spins, the transition is of first order [30,31].

Another subject which has been much studied since the 1980s is the phenomenon called “order by disorder”: we have seen that the GS of frustrated spin systems is highly degenerate and often infinitely degenerate (entropy not zero at temperature T=0). However, it has been shown in many cases that when *T* is turned on the system chooses a state which has the largest entropy, namely the system chooses its order by the largest disorder. We call this phenomenon “order by disorder” or “order by entropic selection” (see references cited in section III. B of Ref. [30]).

We will not discuss these subjects in this review which is devoted to low-dimensional frustrated spin systems.

## 3. Exactly Solved Frustrated Models

**Figure 5:**
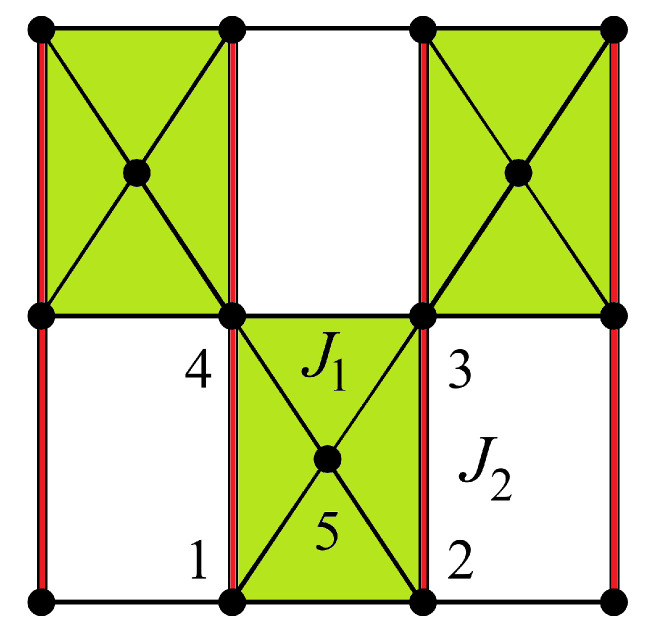
Kagomé lattice: Diagonal and horizontal bonds are nn antiferromagnetic interactions $J_{1}$, vertical double lines indicate the nnn interactions $J_{2}$.

**Figure 6:**
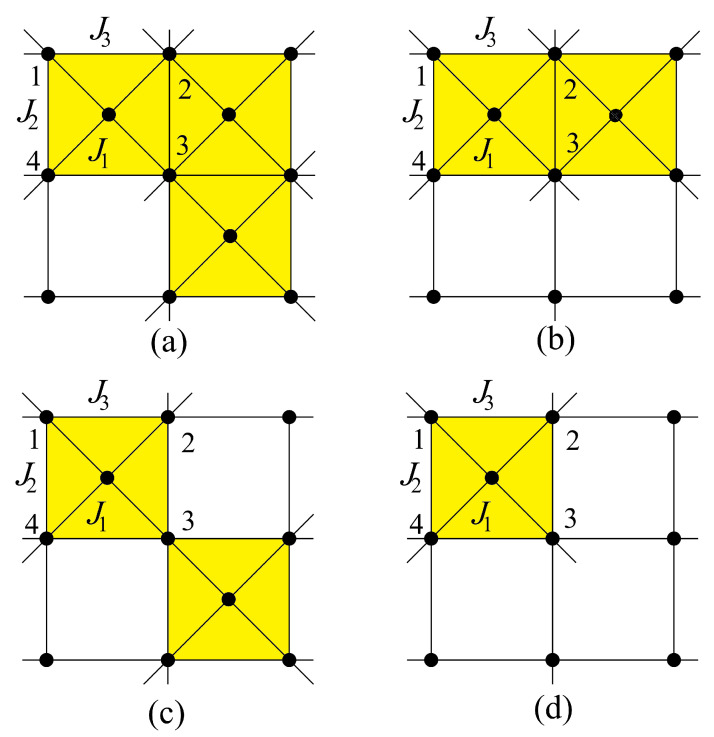
Exactly solved dilute centered square lattices: Interactions along diagonal, vertical and horizontal spin pairs are noted by $J_{1}$, $J_{2}$, and $J_{3}$, respectively.

**Figure 7:**
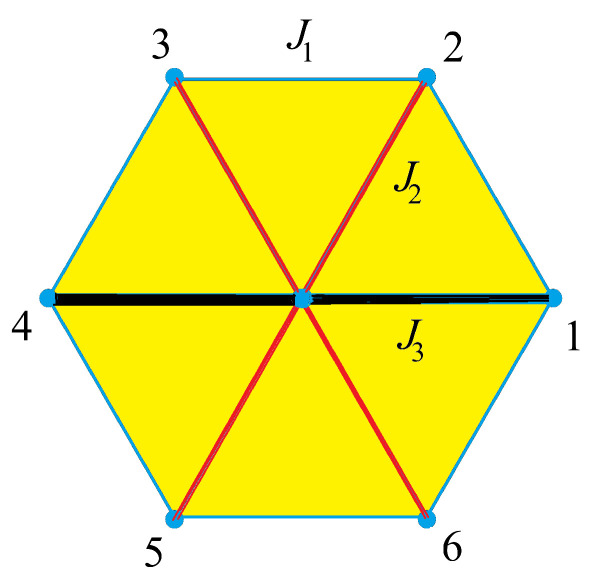
Centered honeycomb lattice. Spins are numbered for decimation demonstration (see text). Blue, red and black bonds denote interactions $J_{1}$, $J_{2}$ and $J_{3}$, respectively.

Any 2D Ising model with non-crossing interactions can be exactly solved. To avoid the calculation of the partition function one can transform the model into a 16-vertex model or a 32-vertex model. The resulting vertex model is exactly solvable. We have applied this method to search for the exact solution of several Ising frustrated 2D models with non-crossing interactions shown in Figure 5, Figure 6 and Figure 7.

Details have been given in Ref. [32]. We outline below a simplified formulation of a model for illustration. The aim is to discuss the results. As we will see these models possess spectacular phenomena due to the frustration.

### 3.1. Example of the Decimation Method

We take the case of the centered honeycomb lattice with the following Hamiltonian

(7) $$H=-J_{1}\sum_{\left(ij\right)}\sigma_{i}\sigma_{j}-J_{2}\sum_{\left(ij\right)}\sigma_{i}\sigma_{j}-J_{3}\sum_{\left(ij\right)}\sigma_{i}\sigma_{j}$$

where $\sigma_{i}=\pm 1$ is an Ising spin at the lattice site *i*. The first, second, and third sums are performed on the spins interacting via $J_{1}$, $J_{2}$ and $J_{3}$ bonds, respectively (see Figure 7). The case $J_{2}=J_{3}=0$ corresponds to the honeycomb lattice, and the case $J_{1}=J_{2}=J_{3}$ to the triangular lattice.

Let σ be the central spin of the lattice cell shown in Figure 7. Other spins are numbered from $\sigma_{1}$ to $\sigma_{6}$. The Boltzmann weight of the elementary cell is written as

(8) $$\begin{array}{ccc} W & = & \exp[K_{1}\left(\sigma_{1}\sigma_{2}+\sigma_{2}\sigma_{3}+\sigma_{3}\sigma_{4}+\sigma_{4}\sigma_{5}+\sigma_{5}\sigma_{6}+\sigma_{6}\sigma_{1}\right)+\\ & & K_{2}\sigma \left(\sigma_{1}+\sigma_{2}+\sigma_{4}+\sigma_{5}\right)+K_{3}\sigma \left(\sigma_{3}+\sigma_{6}\right)] \end{array}$$

where $K_{i}\equiv \frac{J_{i}}{k_{B}T}$ (i=1,2,3). The partition function reads

(9) $$Z=\sum_{\sigma }\prod_{c}W$$

where the sum is taken over all spin configurations and the product over all elementary cells of the lattice. One imposes the periodic boundary conditions. The above model is exactly solvable. To that end, we decimate the central spin of every elementary lattice cell. We finally get a honeycomb Ising model (without centered spins) with multispin interactions.

After decimation of the central spin, namely after summing the values of the central spin σ, the Boltzmann weight of an elementary cell reads

(10) $$\begin{array}{ccc} W^{\prime } & = & 2\exp[K_{1}\left(\sigma_{1}\sigma_{2}+\sigma_{2}\sigma_{3}+\sigma_{3}\sigma_{4}+\sigma_{4}\sigma_{5}+\sigma_{5}\sigma_{6}+\sigma_{6}\sigma_{1}\right)]\times \\ & & \cosh[K_{2}\left(\sigma_{1}+\sigma_{2}+\sigma_{4}+\sigma_{5}\right)+K_{3}\left(\sigma_{3}+\sigma_{6}\right)] \end{array}$$

We show below that this model is in fact a case of the 32-vertex model on the triangular lattice which has an exact solution.

**Figure 8:**
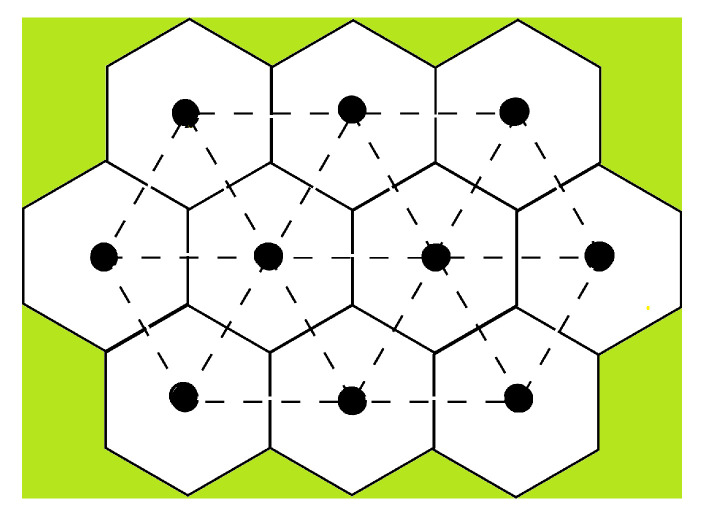
The dual triangular lattice, shown by discontinued lines, of the honeycomb lattice.

We consider the dual triangular lattice of the honeycomb lattice obtained above [33]. The sites of the dual triangular lattice are at the center of each elementary honeycomb cell with bonds perpendicular to the honeycomb ones, as illustrated in Figure 8.

**Figure 9:**
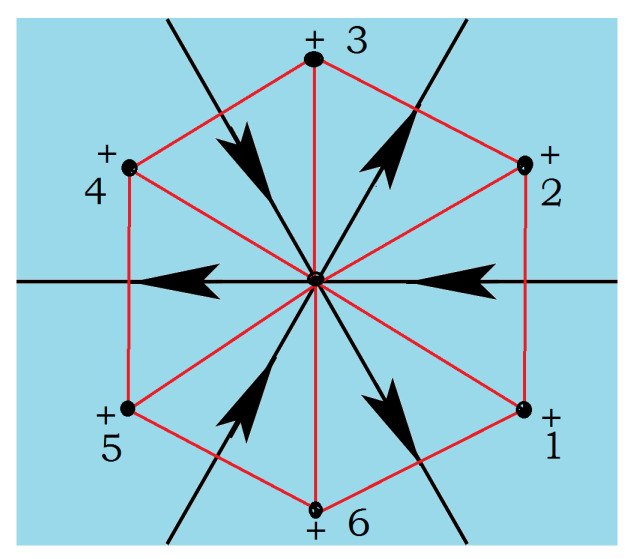
The conventional definition of the “standard” arrows for nn around a site of the triangular lattice: spins are numbered so the arrows can be recognized in examples shown in Figure 10. Please note that the configuration of all down spins has the same arrow configuration. See text.

Let us define the conventional arrow configuration for each site of the dual triangular lattice: if all six spins of the honeycomb cell are parallel, then the arrows, called “standard configuration”, are shown in Figure 9. From this “conventional” configuration, antiparallel spin pairs on the two sides of a triangular lattice bond will have its corresponding arrow change the direction.

**Figure 10:**
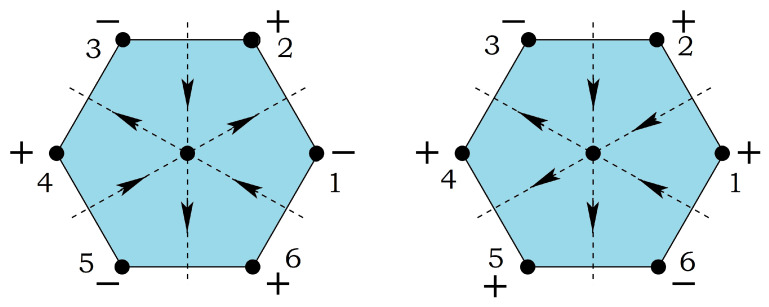
Two examples of spin configurations and their corresponding arrow configurations. To understand, compare with the standard arrows defined in Figure 9. See text.

As examples, two spin configurations on the honeycomb lattice and their corresponding arrow configurations on the triangular lattice are displayed in Figure 10.

Counting all arrow configurations, we obtain 32. To each of these 32 vertices one associates the Boltzmann weight $W^{\prime }\left(\sigma_{1},\sigma_{2},\sigma_{3},\sigma_{4},\sigma_{5},\sigma_{6}\right)$ given by Equation (10). Let us give explicitly a few of them:

(11) $$\begin{array}{ccc} \omega_{1} & = & W^{\prime }\left(+,-,-,-,+,+\right)=2e^{2K_{1}}\; \end{array}$$

(12) $$\begin{array}{ccc} \omega_{2} & = & W^{\prime }\left(+,+,-,+,+,-\right)=2e^{-2K_{1}}\cosh\left(4K_{2}-2K_{3}\right) \end{array}$$

(13) $$\begin{array}{ccc} \omega_{3} & = & W^{\prime }\left(+,-,+,-,+,+\right)=2e^{-2K_{1}}\cosh\left(2K_{3}\right)\; \end{array}$$

(14) $$\begin{array}{ccc} \omega_{4} & = & W^{\prime }\left(+,+,+,+,+,-\right)=2e^{2K_{1}}\cosh\left(4K_{2}\right)\;\\ & & ..... \end{array}$$

Using the above expressions of the 32-vertex model, one finds the following equation for the critical temperature (see details in Ref. [34]):

(15) $$\begin{array}{c} e^{2K_{1}}+e^{-2K_{1}}\cosh\left(4K_{2}-2K_{3}\right)\\ +2e^{-2K_{1}}\cosh\left(2K_{3}\right)+2e^{2K_{1}}+\\ e^{6K_{1}}\cosh\left(4K_{2}+2K_{3}\right)+e^{-6K_{1}}=2\max\{e^{2K_{1}}+\\ e^{-2K_{1}}\cosh\left(4K_{2}-2K_{3}\right)\;\;;\;\;\\ e^{2K_{1}}+e^{-2K_{1}}\cosh\left(2K_{3}\right);e^{6K_{1}}\cosh\left(4K_{1}+2K_{3}\right)+e^{-6K_{1}}\} \end{array}$$

The solutions of this equation are given in Section 3.3.2 below for some special cases.

Following the case studied above, we can study the 2D models shown in Figure 5 and Figure 6: after decimation of the central spin in each square, these models can be transformed into a special case of the 16-vertex model which yields the exact solution for the critical surface (see details in Ref. [32]).

Before showing some results in the space of interaction parameters, let us introduce the definitions of disorder line and reentrant phase.

### 3.2. Disorder Line, Reentrance

It is not the purpose of this review to enter technical details. We would rather like to describe the physical meaning of the disorder line and the reentrance. A full technical review has been given in Ref. [32].

Disorder solutions exist in the paramagnetic region which separate zones of fluctuations of different nature. They are where the short-range pre-ordering correlations change their nature to allow for transitions in the phase diagrams of anisotropic models. They imply constraints on the analytical behavior of the partition function of these models.

To obtain the disorder solution one makes a certain local decoupling of the degrees of freedom. This yields a dimension reduction: a 2D system then behaves on the disorder line as a 1D system. This local decoupling is made by a simple local condition imposed on the Boltzmann weights of the elementary cell [35,36,37].

This is very important while interpreting the system behavior: on one side of the disorder line, pre-ordering fluctuations have correlation different from those of the other side. Crossing the line, the system pre-ordering correlation changes. The dimension reduction is often necessary to realize this.

Please note that disorder solutions may be used in the study of cellular automata as it has been shown in Ref. [38].

Let us give now a definition for the reentrance. A reentrant phase lies between two ordered phases. For example, at low temperature (*T*) the system is in an ordered phase I. Increasing *T*, it undergoes a transition to a paramagnetic phase *R*, but if one increases further *T*, the system enters another ordered phase II before becoming disordered at a higher *T*. Phase *R* is thus between two ordered phases I and II. It is called “reentrant paramagnetic phase” or “reentrant phase”.

How physically is it possible? At a first sight, it cannot be possible because the entropy of an ordered phase is smaller than that of a disordered phase so that the disordered phase *R* cannot exist at lower *T* than the ordered phase II. In reality, as we will see below, phase II has always a partial disorder which compensates for the loss of entropy while going from *R* to II. The principle that entropy increases with *T* is thus not violated.

### 3.3. Phase Diagram

#### 3.3.1. Kagomé Lattice

The Kagomé lattice shown in Figure 5 has attracted much attention not only by its great interest in statistical physics but also in real materials [17]. The Kagomé Ising model with only nn interaction $J_{1}$ has been solved a long time ago [39]. No phase transition at finite *T* when $J_{1}$ is antiferromagnetic. Taking into account the nnn interaction $J_{2}$, we have solved [40] this model by transforming it into a 16-vertex model which satisfies the free-fermion condition. The equation of the critical surface is

(16) $$\begin{array}{ccc} \frac{1}{2}\;\left[\exp\left(2K_{1}+2K_{2}\right)\cosh\left(4K_{1}\right)+\exp\left(-2K_{1}-2K_{2}\right)\right] & + & \\ \cosh\left(2K_{1}-2K_{2}\right)+2\cosh\left(2K_{1}\right)=2\max\{\frac{1}{2}\;[\exp(2K_{1} & + & 2K_{2})\cosh\left(4K_{1}\right)+\\ \exp\left(-2K_{1}-2K_{2}\right)]\;;\;\cosh\left(2K_{2}-2K_{1}\right) & ; & \cosh(2K_{1})\} \end{array}$$

**Figure 11:**
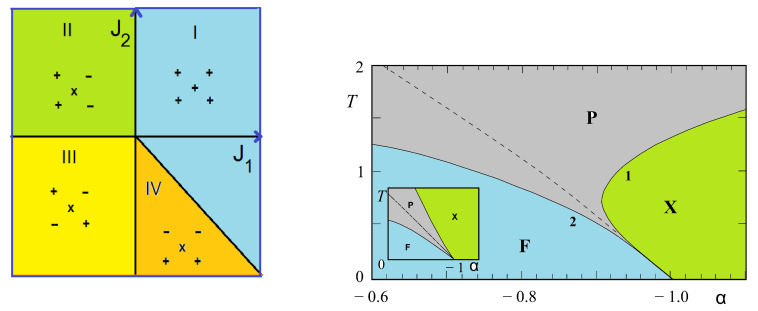
Left: Each color represents a ground-state configuration in the space ($J_{1},J_{2})$ where +, −, and *x* denote up, down, and free spins, respectively. Right: Phase diagram in the space ($\alpha =J_{2}/J_{1},T$) with $J_{1}>0$. *T* is in the unit of $J_{1}/k_{B}$. Solid lines are critical lines, dashed line is the disorder line. P, F, and X stand for paramagnetic, ferromagnetic and partially disordered phases, respectively. The inset shows schematically the enlarged region near the critical value $J_{2}/J_{1}=-1$.

We are interested in the region near the phase boundary between two phases IV (partially disordered) and I (ferromagnetic) in Figure 11 (left). We show in Figure 11 (right) the small region near the boundary $\alpha =J_{2}/J_{1}=-1$ which has the reentrant paramagnetic phase and a disorder line.

We note that only near the phase boundary such a reentrant phase and a disorder line can exist.

If we suppose that all interactions $J_{1}$, $J_{2}$ and $J_{3}$ in the model shown in Figure 5 are different, the phase diagram becomes very rich [41]. For instance, the reentrance can occur in an *infinite region* of interaction parameters and several reentrant phases can occur for a given set of interactions when *T* varies.

The Hamiltonian reads

(17) $$H=-J_{1}\sum_{\left(ij\right)}\sigma_{i}\sigma_{j}-J_{2}\sum_{\left(ij\right)}\sigma_{i}\sigma_{j}-J_{3}\sum_{\left(ij\right)}\sigma_{i}\sigma_{j}$$

where $\sigma_{i}$ is the Ising spin occupying the lattice site *i*, and the sums are performed over the spin pairs connected by $J_{1}$, $J_{2}$ and $J_{3}$, respectively.

**Figure 12:**
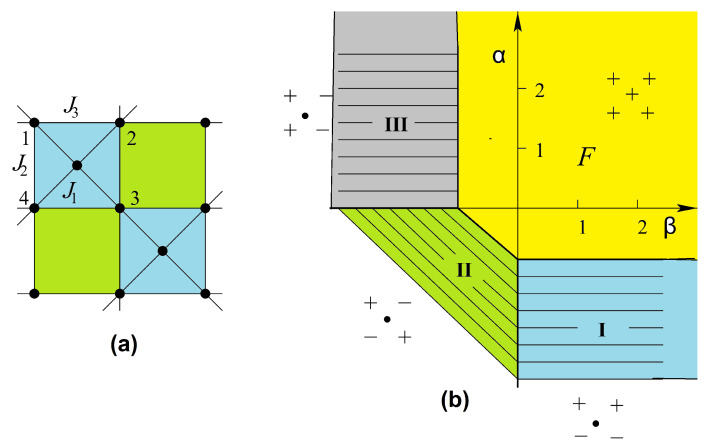
(**a**) Generalized Kagomé lattice: $J_{1}$, $J_{2}$ and $J_{3}$ denote the diagonal, vertical and horizontal bonds, respectively. (**b**) The ground-state phase diagram in the space ($\alpha =J_{2}/J_{1},\beta =J_{3}/J_{1}$). Each phase is displayed by a color with up, down, and free spins denoted by +, −, and o, respectively. I, II, III, and F indicate the three partially disordered phases and the ferromagnetic phase, respectively.

The phase diagram at temperature T=0 is shown in Figure 12 in the space ($\alpha =J_{2}/J_{1}$, $\beta =J_{3}/J_{1}$), supposing $J_{1}>0$. The spin configuration of each phase is indicated. The three partially disordered phases (I, II, and III) have free central spins. With $J_{1}<0$, it suffices to reverse the central spin in the F phase of Figure 12. In addition, the permutation of $J_{2}$ and $J_{3}$ will not change the system, because it is equivalent to a π/2 rotation of the lattice.

We examine now the temperature effect. We have seen above that a partially disordered phase lies next to the ferromagnetic phase in the GS gives rise to the reentrance phenomenon. We expect therefore similar phenomena near the phase boundary in the present model. As it turns out, we find below a new and richer behavior of the phase diagram.

We use the decimation of central spins described in Ref. [32], we get then a checkerboard Ising model with multispin interactions. This corresponds to a symmetric 16-vertex model which satisfies the free-fermion condition [42,43,44]. The critical temperature is the solution of the following equation

(18) $$\begin{array}{c} \cosh\left(4K_{1}\right)\exp\left(2K_{2}+2K_{3}\right)+\exp\left(-2K_{2}-2K_{3}\right)\\ =2\cosh\left(2K_{3}-2K_{2}\right)\pm 4\cosh\left(2K_{1}\right) \end{array}$$

**Figure 13:**
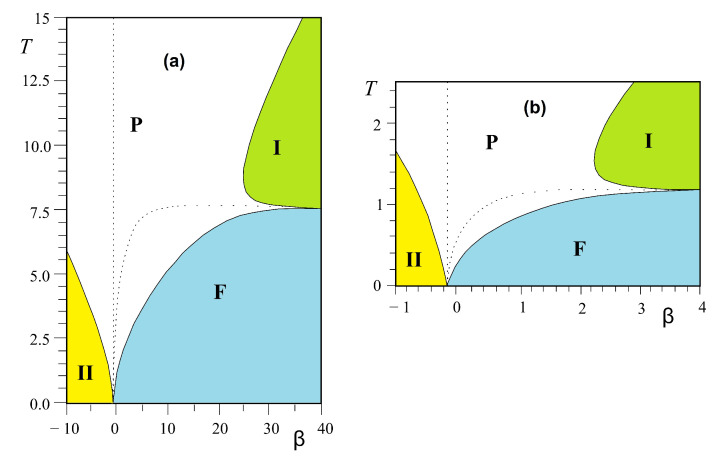
Phase diagram in the ($\beta =J_{3}/J_{1},T$) space for negative $\alpha =J_{2}/J_{1}$. (**a**) α=−0.25; (**b**) α=−0.8. Partially disordered phases of type I and II and F are defined in Figure 12. The disorder lines are shown by dotted lines.

Note the invariance of Equation (18) with respect to changing $K_{1}\to -K_{1}$ and interchanging $K_{2}$ and $K_{3}$. Let us show just the solution near the phase boundary in the plane ($\beta =J_{3}/J_{1},T$) for two values of $\alpha =J_{2}/J_{1}$. It is interesting to note that in the interval 0>α>−1, there exist three critical lines. Two of them have a common horizontal asymptote as β tends to infinity. They limit a reentrant paramagnetic phase between the F phase and the partially disordered phase I for β between $\beta_{2}$ and infinite β (see Figure 13). Such an infinite reentrance has never been found before in other models. With decreasing α, $\beta_{2}$ tends to zero and the F phase is reduced (comparing Figure 13a,b). For α<−1, the F phase and the reentrance no longer exist.

We note that for −1<α<0, the model possesses two disorder lines (see equations in Ref. [41]) starting from a point near the phase boundary β=−1 for α close to zero; this point position moves to β=0 as α tends to −1 (see Figure 13).

#### 3.3.2. Centered Honeycomb Lattice

We use the decimation of the central spin of each elementary cell as shown in Section 3.1. After the decimation, we obtain a model equivalent to a special case of the 32-vertex model [45] on a triangular lattice which satisfies the free-fermion condition. The general treatment has been given in Ref. [34]. Here we show the result of the case where $K_{2}=K_{3}$. Equation (15) is reduced to

(19) $$\exp\left(3K_{1}\right)\cosh\left(6K_{2}\right)+\exp\left(-3K_{1}\right)=3\left[\exp\left(K_{1}\right)+\exp\left(-K_{1}\right)\cosh\left(2K_{2}\right)\right]$$

When $K_{2}=0$, Equation (15) gives the critical line

(20) $$\exp\left(3K_{1}\right)\cosh\left(2K_{3}\right)+\exp\left(-3K_{1}\right)=3\left[\exp\left(K_{1}\right)+\exp\left(-K_{1}\right)\cosh\left(2K_{3}\right)\right]$$

When $K_{3}=0$, we observe a reentrant phase. The critical lines are given by

(21) $$\cosh\left(4K_{2}\right)=\frac{\exp\left(4K_{1}\right)+2\exp\left(2K_{1}\right)+1}{\left[1-\exp\left(4K_{1}\right)\right]\exp\left(2K_{1}\right)}$$

(22) $$\cosh\left(4K_{2}\right)=\frac{3\exp\left(4K_{1}\right)+2\exp\left(2K_{1}\right)-1}{\left[\exp\left(4K_{1}\right)-1\right]\exp\left(2K_{1}\right)}$$

**Figure 14:**
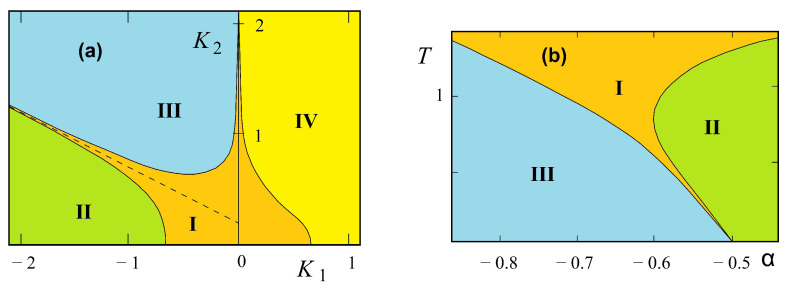
Centered honeycomb lattice: (**a**) Phase diagram in the space ($K_{1},K_{2}$), discontinued line is the asymptote; (**b**) Reentrance in the space ($T,\alpha =K_{2}/K_{1}$). I, II, III phases denote paramagnetic, partially disordered and ordered phases, respectively.

The phase diagram obtained from Equations (21) and (22) near the phase boundary α=−0.5 is displayed in Figure 14. One observes here that the reentrant zone goes down to T=0 at the boundary α=−0.5 separating the GS phases II and III (see Figure 14b).

Please note that phase II has the antiferromagnetic ordering on the hexagon and the central spin free to flip, while phase III is the ordered phase where the central spin is parallel to 4 diagonal spins (see Figure 2 of Ref. [34]). Therefore, if −0.6<α<−0.5 (reentrant region, see Figure 14b), when one increases *T* from T=0, ones goes across successively the ordered phase III, the narrow paramagnetic reentrant phase and the partially disordered phase II. Two remarks are in order: (i) The reentrant phase occurs here between an ordered phase and a partially disordered phase. However, as will be seen below, we discover in the three-center square lattice, reentrance can occur between two partially disordered phase; (ii) In any case, we find reentrance between phases when and only when there are free spins in the GS. The entropy of the high-*T* partially disordered phase is higher than that of the low-*T* one. The second thermodynamic principle is not violated.

It is noted that the present honeycomb model does not possess a disorder solution with a reduction of dimension as the Kagomé lattice shown earlier.

#### 3.3.3. Centered Square Lattices

In this paragraph, we study several centered square Ising models by mapping them onto 8-vertex models that satisfy the free-fermion condition. The exact solution is then obtained for each case. Let us anticipate that in some cases, for a given set of parameters, up to five transitions have been observed with varying temperature. In addition, there are two reentrant paramagnetic phases going to infinity in the space of interaction parameters, and there are two additional reentrant phases found, each in a small zone of the phase space [46,47].

We consider the dilute centered square lattices shown in Figure 6. The Hamiltonian of these models reads

(23) $$H=-J_{1}\sum_{\left(ij\right)}\sigma_{i}\sigma_{j}-J_{2}\sum_{\left(ij\right)}\sigma_{i}\sigma_{j}-J_{3}\sum_{\left(ij\right)}\sigma_{i}\sigma_{j}$$

where $\sigma_{i}$ is an Ising spin at the lattice site *i*. The sums are performed over the spin pairs interacting by $J_{1}$, $J_{2}$ and $J_{3}$ bonds (diagonal, vertical and horizontal bonds, respectively).

**Figure 15:**
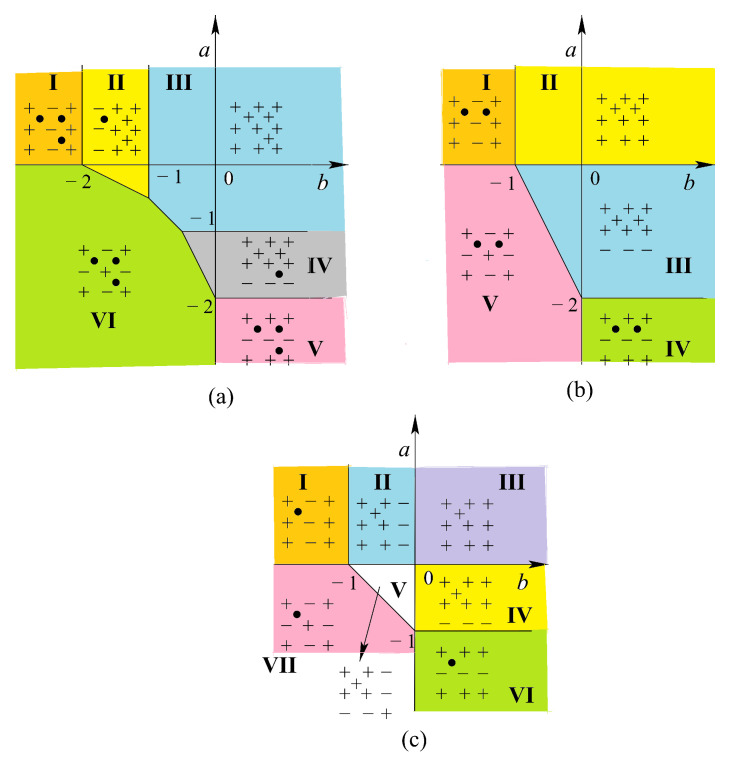
Ground-state phase diagram in the space ($a=J_{2}/J_{1}$, $b=J_{3}/J_{1}$) for (**a**) three-center square lattice; (**b**) two-adjacent center case; (**c**) and one-center case. Phase boundaries are indicated by heavy lines. Each phase is numbered and the spin configuration is displayed (+, −, and o are up, down, and free spins, respectively).

Figure 15 shows the ground-state phase diagrams of the models displayed in Figure 6a,b,d, where $a=J_{2}/J_{1}$ and $b=J_{3}/J_{1}$. The spin configurations in different phases are also displayed. The model in Figure 15a has six phases (numbered from I to VI), five of which (I, II, IV, V and VI) are partially disordered (at least one centered spin being free), the model in Figure 15b has five phases, three of which (I, IV, and V) are partially disordered, and the model in Figure 15c has seven phases with three partially disordered ones (I, VI, and VII).

It is interesting to note that each model shown in Figure 6 possesses the reentrance along most of the phase boundary lines when the temperature is turned on. This striking feature of the centered square Ising lattices has not been observed in other known models.

**Figure 16:**
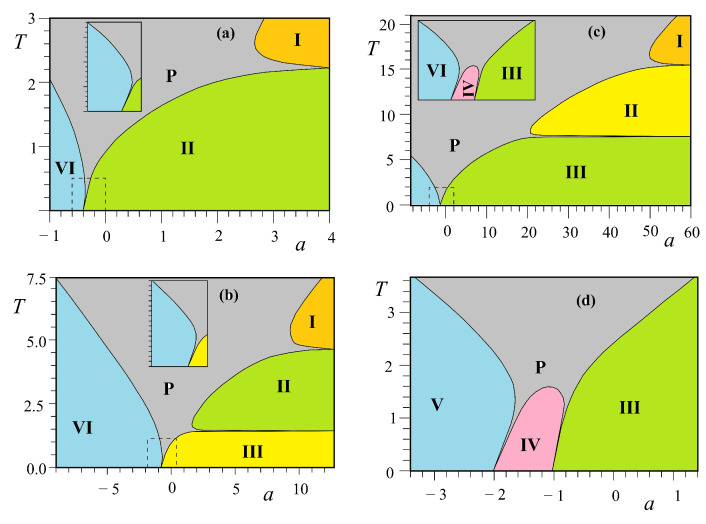
Three-center model: phase diagram in the space ($T,a=J_{2}/J_{1}$) for several values of $b=J_{3}/J_{1}$: (**a**) b=−1.25, (**b**) b=−0.75, (**c**) b=−0.25, (**d**) b=0.75. Reentrant regions indicated by discontinued lines are enlarged in the insets. A number indicates the corresponding spin configuration shown in Figure 15a. P is paramagnetic phase.

Let us show in Figure 16 the results of the three-center model of Figure 6a, in the space ($a=J_{2}/J_{1}$, *T*) for typical values of $b=J_{3}/J_{1}$.

For b<−1, there are two reentrances as seen in Figure 16a for b=−1.25. The phase diagram is shown using the same numbers of corresponding ground-state configurations of Figure 15. Please note that the centered spins disordered at T=0 in phases I, II and VI (Figure 15a) remain so at all *T*. Note also that the reentrance occurs always at a phase boundary. This point is emphasized in this paper through various shown models.

For −1<b<−0.5, there are three reentrant paramagnetic phases as shown in Figure 16b, two of them on the positive *a* are so narrow while *a* goes to infinity. Please note that the critical lines in these regions have horizontal asymptotes. For a large value of *a*, one has five transitions with decreasing *T*: paramagnetic phase–partially disordered phase I—first reentrant paramagnetic phase—partially disordered phase II—second reentrant paramagnetic phase—ferromagnetic phase (see Figure 16b). To our knowledge, a model that exhibits such five phase transitions with two reentrances has never been found before.

For −0.5<b≤0, another reentrance is found for a<−1 as seen in the inset of Figure 16c. With increasing *b*, the ferromagnetic phase III in the phase diagram becomes large, reducing phases I and II. At b=0, only the ferromagnetic phase remains.

For positive *b*, we have two reentrances for a<0, ending at a=−2 and a=−1 when T=0 as seen in Figure 16d.

In conclusion, we summarize that in the three-center square lattice model shown in Figure 6a, we found two reentrant phases occurring on the temperature scale at a given set of interaction parameters. A new feature found here is that a reentrant phase can occur between two partially disordered phases, unlike in other models such as the Kagomé Ising lattice where a reentrant phase occurs between an ordered phase and a partially disordered phase.

### 3.4. Summary and Discussion

The present section shows spectacular phenomena due to the frustration. What to be retained is the fact that those phenomena occur around the boundary of two phases of different GSs, namely different symmetries. These phenomena include

(1) the partial disorder at equilibrium: disorder is not equally shared on all particles as usually the case in unfrustrated systems.
(2) the reentrance: this occurs around the phase boundary when *T* increases → the phase with larger entropy will win at finite *T*. In other words, this is a kind of selection by entropy.
(3) the disorder line: this line occurs in the paramagnetic phase. It separates the pre-ordering zones between two nearby ordered phases.

In the present section, we looked for interesting effects of the frustration by solving exactly several 2D Ising models with non-crossing interactions. This has been done by the decimation method combined with the mapping to vertex models. We know that vertex models are exactly solvable when the free-fermion conditions are satisfied. This is the case in the 8-, 16-, and 32-vertex models shown above. The striking results mentioned above, namely the partial disorder, the reentrance, the disorder line and the multiple phase transitions, are expected to exist in models other than the Ising model and in three-dimensional lattices, although they cannot be exactly solved. We mention that partial disorder in some 3D highly frustrated Ising systems has been found: for instance, the fully frustrated simple cubic lattice [48,49], a stacked triangular Ising antiferromagnet [50,51] and a body-centered cubic (bcc) crystal [52]. For non-Ising spins such as quantum spins, partial disorder has also been found [53,54,55]. As for the reentrance in 3D, we mention the case of a special lattice which is exactly solved [56]. We believe that reentrance should also exist in the phase space of many other 3D systems. We found for example numerical evidence of a reentrance for the bcc Ising case [52] and a frustrated XY model on stacked 3D checkerboard lattices [55].

Please note that evidence of a reentrance has been found for the *q*-state Potts model on the 2D frustrated Villain lattice [57,58].

Finally, through the examples shown above, we see that for the reentrance to occur it is necessary to have free spins in the GS.

## 4. Physics of Thin Films: Surface Magnetism, Background

### 4.1. Surface Parameters

Surface physics has been rapidly developed in the last 30 years thanks to the progress in the fabrication and the characterization of films of very thin thickness down to a single atomic layer. A lot of industrial applications have been made in memory storage, magnetic sensors, …using properties of thin films.

Theory and simulation have also been in parallel developed to understand these new properties and to predict further interesting effects. In the following we introduce some useful microscopic mechanisms which help understand macroscopic effects observed in experiments.

The existence of a surface on a crystal causes a lot of modifications at the microscopic levels. First, the lack of neighbors of atoms on the surface causes modifications in their electronic structure giving rise to modifications in electron orbital and atomic magnetic moment by for example the spin-orbit coupling and in interaction parameters with neighboring atoms (exchange interaction, for example). In addition, surfaces can have impurities, defects (vacancies, islands, dislocations, …). In short, we expect that the surface parameters are different from the bulk ones. Consequently, we expect physical properties at and near a surface are different from those in the bulk. For the fundamental theory of magnetism and its application to surface physics, the reader is referred to Ref. [6].

In the following we outline some principal microscopic mechanisms which dominate properties of magnetic thin films.

### 4.2. Surface Spin Waves: Simple Examples

In magnetically ordered systems, spin-wave (SW) excitations dominate thermodynamic properties at low *T*. The presence of a surface modifies the SW spectrum. We show below that it gives rise to SW modes localized near the surface. These modes lie outside the bulk SW spectrum and modify the low-*T* behavior of thin films.

Let us calculate these modes in some simple cases. We give below for pedagogical purpose some technical details.

We consider a thin film of $N_{T}$ layers stacked in the *z* direction. The Hamiltonian is written as

(24) $$\begin{array}{ccc} H & = & -2\sum_{<i,j>}J_{ij}S_{i}\cdot S_{j}-2\sum_{<i,j>}D_{ij}S_{i}^{z}S_{j}^{z}\\ & = & -2\sum_{\langle i,j\rangle }J_{ij}\left(S_{i}^{z}S_{j}^{z}+\frac{1}{2}\left(S_{i}^{+}S_{j}^{-}+S_{i}^{-}S_{j}^{+}\right)\right)-2\sum_{<i,j>}D_{ij}S_{i}^{z}S_{j}^{z} \end{array}$$

where $J_{ij}$ is the exchange interaction between two nn Heisenberg quantum spins, and $D_{ij}>0$ denotes an exchange anisotropy. $S_{j}^{+}$ and $S_{j}^{-}$ are the standard spin operators $S_{j}^{\pm }=S_{j}^{x}\pm iS_{j}^{y}$.

For simplicity, we suppose no crystalline defects and no impurities at the surface and all interactions are identical for surface and bulk spins. It is known that in perfect crystals the spin waves dominate low-temperature properties [6]. In a thin film, there often exist SW modes localized near the surface. Such surface spin waves are at the origin of the low surface magnetization and transition temperature. One can calculate the SW energy using the method of equation of motion, the Holstein-Primakoff method and the Green’s function method. Here we use for illustration the Green’s function method which the author has developed for thin films (see details in Ref. [59,60]). This method shall be generalized below for helimagnets and other systems with non-collinear spin configurations.

Let us define the double-time Green’s function by

(25) $$G_{i,j}\left(t,t^{\prime }\right)=\langle \langle S_{i}^{+}\left(t\right);S_{j}^{-}\left(t^{\prime }\right)\rangle \rangle$$

The equation of motion of $G_{i,j}\left(t,t^{\prime }\right)$ is written as

(26) $$i\hbar \frac{dG_{i,j}\left(t,t^{\prime }\right)}{dt}={\left(2\pi \right)}^{-1}\langle \left[S_{i}^{+}\left(t\right),S_{j}^{-}\left(t^{\prime }\right)\right]\rangle +\langle \langle \left[S_{i}^{+},H\right]\left(t\right);S_{j}^{-}\left(t^{\prime }\right)\rangle \rangle$$

where […] denotes the boson commutator and 〈…〉 the canonical thermal average given by

(27) $$\langle F\rangle ={\mathrm{Tre}}^{-\beta H}F/{\mathrm{Tre}}^{-\beta H}$$

with $\beta =1/k_{B}T$. When we perform the commutator of Equation (26), we obtain Green’s functions of higher orders. These functions can be reduced by the use of the Tyablikov approximation [61]

(28) $$\langle \langle S_{m}^{z}S_{i}^{+};S_{j}^{-}\rangle \rangle ≃\langle S_{m}^{z}\rangle \langle \langle S_{i}^{+};S_{j}^{-}\rangle \rangle ,$$

Thus, we get the same kind of Green’s function defined in Equation (25).

In a thin film, the system is supposed to be infinite in the xy plane, we can therefore use the in-plane Fourier transforms

(29) $$G_{i,j}\left(t,t^{\prime }\right)=\frac{1}{\Delta }\int \int dk_{xy}\frac{1}{2\pi }\int_{-\infty }^{+\infty }d\omega \;e^{-i\omega (t-t^{\prime })}\;g_{n,n^{\prime }}\left(\omega ,k_{xy}\right)\;e^{ik_{xy}.\left(R_{i}-R_{j}\right)}$$

Here, ω denotes the magnon frequency and $k_{xy}$ the wave vector parallel to the surface. The position of the spin at the site *i* is $R_{i}$. *n* and $n^{\prime }$ are respectively the planes to which *i* and *j* belong (n=1 is the index of the surface). Please note that the integration on $k_{xy}$ is performed in the first Brillouin zone of surface Δ in the xy plane.

Equation (26) yields

(30) $$\left(\hbar \omega -A_{n}\right)g_{n,n^{\prime }}+B_{n}\left(1-\delta_{n,1}\right)g_{n-1,n^{\prime }}+C_{n}\left(1-\delta_{n,N_{T}}\right)g_{n+1,n^{\prime }}=2\delta_{n,n^{\prime }}<S_{n}^{z}>$$

The coefficients $A_{n}$, $B_{n}$ and $C_{n}$ depend on the crystalline structure of the film, for instance:

- Film of simple cubic lattice
  (31) $$\begin{array}{ccc} A_{n} & = & -2J_{n}<S_{n}^{z}>C\gamma_{k}+2C\left(J_{n}+D_{n}\right)<S_{n}^{z}>\\ & & +2\left(J_{n,n+1}+D_{n,n+1}\right)<S_{n+1}^{z}>\\ & & +2\left(J_{n,n-1}+D_{n,n-1}\right)<S_{n-1}^{z}> \end{array}$$
  (32) $$\begin{array}{ccc} B_{n} & = & 2J_{n,n-1}<S_{n}^{z}> \end{array}$$
  (33) $$\begin{array}{ccc} C_{n} & = & 2J_{n,n+1}<S_{n}^{z}> \end{array}$$
  where C=4 (in-plane coordination number) and $\gamma_{k}=\frac{1}{2}\left[\cos\left(k_{x}a\right)+\cos\left(k_{y}a\right)\right]$.
- Film of body-centered cubic lattice
  (34) $$\begin{array}{ccc} A_{n} & = & 8\left(J_{n,n+1}+D_{n,n+1}\right)<S_{n+1}^{z}>\\ & & +8\left(J_{n,n-1}+D_{n,n-1}\right)<S_{n-1}^{z}> \end{array}$$
  (35) $$\begin{array}{ccc} B_{n} & = & 8J_{n,n-1}<S_{n}^{z}>\gamma_{k} \end{array}$$
  (36) $$\begin{array}{ccc} C_{n} & = & 8J_{n,n+1}<S_{n}^{z}>\gamma_{k} \end{array}$$
  where $\gamma_{k}=\cos\left(k_{x}a/2\right)\cos\left(k_{y}a/2\right)$

Using Equation (30) for n=1, 2,…, $N_{T}$, we get $N_{T}$ coupled equations which is written in a matrix equation

(37) M(ω)g=u

where u is a column matrix whose n-th element is $2\delta_{n,n^{\prime }}<S_{n}^{z}>$.

For each $k_{xy}$ we can calculate the magnon energy $\hbar \omega (k_{xy})$ by solving the secular equation det|M|=0. This gives $N_{T}$ values of $\hbar \omega_{i}$ ($i=1,...,N_{T}$). We note that $\omega_{i}$ depends on all $\langle S_{n}^{z}\rangle$ contained in the coefficients $A_{n}$, $B_{n}$ and $C_{n}$.

The magnetization $\langle S_{n}^{z}\rangle$ of the layer *n* in the case where $S=\frac{1}{2}$ is calculated by (see chapter 6 of Ref. [6]):

(38) $$\langle S_{n}^{z}\rangle =\frac{1}{2}-\langle S_{n}^{-}S_{n}^{+}\rangle$$

where $\langle S_{n}^{-}S_{n}^{+}\rangle$ is given by the following spectral theorem

(39) $$\begin{array}{ccc} \langle S_{i}^{-}S_{j}^{+}\rangle & = & \lim_{\epsilon \to 0}\frac{1}{\Delta }\int \int dk_{xy}\int_{-\infty }^{+\infty }\frac{i}{2\pi }\left[g_{n,n^{\prime }}\left(\omega +i\epsilon \right)-g_{n,n^{\prime }}\left(\omega -i\epsilon \right)\right]\\ & & \times \frac{d\omega }{e^{\beta \omega }-1}e^{ik_{xy}.\left(R_{i}-R_{j}\right)}. \end{array}$$

where $\epsilon =0^{+}$ is a very small constant. Equation (38) becomes

(40) $$\langle S_{n}^{z}\rangle =\frac{1}{2}-\lim_{\epsilon \to 0}\frac{1}{\Delta }\int \int dk_{xy}\int_{-\infty }^{+\infty }\frac{i}{2\pi }\left[g_{n,n}\left(\omega +i\epsilon \right)-g_{n,n}\left(\omega -i\epsilon \right)\right]\frac{d\omega }{e^{\beta \hbar \omega }-1}$$

where the Green’s function $g_{n,n}$ is given by the solution of Equation (37)

(41) $$g_{n,n}=\frac{{\left|M\right|}_{n}}{\left|M\right|}$$

${\left|M\right|}_{n}$ is the determinant obtained by replacing the *n*-th column of |M| by u.

To simplify we write $\hbar \omega_{i}=E_{i}$ and ℏω=E hereafter. We factorize

(42) $$\left|M\right|=\prod_{i}\left(E-E_{i}\right)$$

using $E_{i}$ ($i=1,\ldots ,N_{T}$), the poles of the Green’s function. $g_{n,n}$ is rewritten as

(43) $$g_{n,n}=\sum_{i}\frac{f_{n}\left(E_{i}\right)}{E-E_{i}}$$

where $f_{n}\left(E_{i}\right)$ is given by

(44) $$f_{n}\left(E_{i}\right)=\frac{{\left|M\right|}_{n}\left(E_{i}\right)}{\prod_{j\neq i}\left(E_{i}-E_{j}\right)}$$

With Equations (40) and (43) and

(45) $$\frac{1}{x-i\eta }-\frac{1}{x+i\eta }=2\pi i\delta \left(x\right)$$

we get

(46) $$\langle S_{n}^{z}\rangle =\frac{1}{2}-\frac{1}{\Delta }\int \int dk_{x}dk_{y}\sum_{i=1}^{N_{T}}\frac{f_{n}\left(E_{i}\right)}{e^{\beta E_{i}}-1}$$

where $n=1,...,N_{T}$.

Since $<S_{n}^{z}>$ depends on the neighboring magnetizations, we should solve by iteration the Equation (46) written for $n=1,\ldots ,N_{T}$ to get the layer magnetizations at *T*.

The critical temperature $T_{c}$ is calculated self-consistently using the Equation (46), with all $<S_{n}^{z}>$ tending to zero.

**Figure 17:**
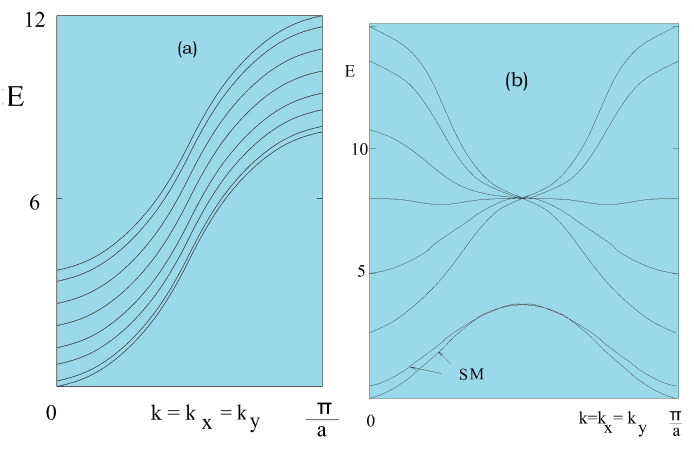
(**a**) Spin-wave energy E=ℏω of the simple cubic ferromagnetic film as a function of $k\equiv k_{x}=k_{y}$ for $N_{T}=8$ and D/J=0.01, no surface mode is found; (**b**) Spin-wave energy for the body-centered cubic film with the same parameter. Surface modes are indicated by SM.

We show in Figure 17a the SW spectrum of a simple cubic film where there is no surface SW mode. Figure 17b shows the case of a body-centered cubic ferromagnetic case where there are two branches of surface localized modes.

Please note that a surface mode has a damping SW amplitude when going from the surface to the interior. The SW amplitudes for each mode are in fact their eigenvectors calculated from Equation (41). Since acoustic surface localized spin waves have low energies, integrands on the right-hand side of Equation (46) are large, making $<S_{n}^{z}>$ to be small and causing a diminution of $T_{c}$ in thin films.

**Figure 18:**
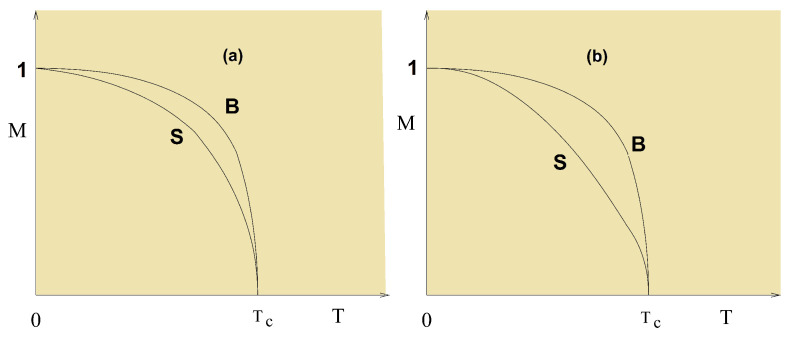
Surface and 2nd layer magnetizations (*S* and *B* curves, respectively) versus *T* for films of (**a**) simple cubic and (**b**) body-centered cubic lattices. $N_{T}=4$, D=0.01J, J=1.

We show in Figure 18 the first- and second-layer magnetizations versus *T* in the films shown above using $N_{T}=4$.

Calculations for antiferromagnetic thin films and other cases with non-collinear spin configurations can be performed using generalized Green’s functions [59,60,62] with the general Hamiltonian defined for two spins $S_{i}$ and $S_{j}$ forming an angle $\cos\theta_{ij}$: one can write the Hamiltonian in the local coordinates as follows [63]

(47) $$\begin{array}{ccc} H & = & -\sum_{<i,j>}J_{i,j}\{\frac{1}{4}\left(\cos\theta_{ij}-1\right)\left(S_{i}^{+}S_{j}^{+}+S_{i}^{-}S_{j}^{-}\right)\\ & + & \frac{1}{4}\left(\cos\theta_{ij}+1\right)\left(S_{i}^{+}S_{j}^{-}+S_{i}^{-}S_{j}^{+}\right)\\ & + & \frac{1}{2}\sin\theta_{ij}\left(S_{i}^{+}+S_{i}^{-}\right)S_{j}^{z}-\frac{1}{2}\sin\theta_{ij}S_{i}^{z}\left(S_{j}^{+}+S_{j}^{-}\right)\\ & + & \cos\theta_{ij}S_{i}^{z}S_{j}^{z}\}-\sum_{<i,j>}I_{i,j}S_{i}^{z}S_{j}^{z}\cos\theta_{ij} \end{array}$$

where an anisotropy (last term) is added for numerical convergence at long-wave lengths. This term is necessary for very thin film thickness since it is known that there is no ordering for isotropic Heisenberg spins in strictly 2D at finite temperatures [64].

The angles between nn spins in the GS are calculated by minimizing the interaction energy with respect to interaction parameters [65,66]. Replacing the angle values in the Hamiltonian, and follow the steps presented above for the collinear case, one then gets a matrix which can be numerically diagonalized to obtain the SW spectrum. Other physical properties can be self-consistently calculated using the SW spectrum as for the collinear spin configuration.

## 5. Frustrated Thin Films: Surface Phase Transition

Having given the background in the previous section, we can show some results here. The reader is referred to the original papers for details. Our aim here is to discuss physical effects due to the conditions of the surface.

As said earlier, the combination of the frustration and the surface effect gives rise to drastic effects. This is seen in the examples shown in the following.

The effects of surface anisotropies and dipole-dipole interactions have been treated in some of our earlier works. However, to keep the length of the present review reasonable, we do not discuss them here. The reader is referred to Ref. [67] for the re-orientation transition in molecular thin films for the Potts model with dipolar interaction in competition with the film perpendicular anisotropy. The same problem was studied with the Heisenberg spin model in Ref. [68]. Please note that in these works, evidence of the reentrance is found near the GS phase boundary between the in-plane spin configuration and the perpendicular one.

### 5.1. Frustrated Surfaces

We show here the case of a ferromagnetic film with frustrated surfaces [65], using the analytical Green’s function method and extensive Monte Carlo simulations. Effects of frustrated surfaces on the properties of a ferromagnetic thin film are presented.

The system is made by stacking triangular layers of Heisenberg spins in the *z* direction. The in-plane surface interaction $J_{s}$ can be antiferromagnetic or ferromagnetic. All other interactions are ferromagnetic. We show that the ground-state spin configuration is non-collinear when $J_{s}$ is lower than a critical value $J_{s}^{c}$. The film surfaces are then frustrated. We anticipate here that in this case, there are two phase transitions, one for the disordering of the surface and the other for the disordering of the interior layers. As seen below, good agreement between Monte Carlo and Green’s function results are achieved.

### 5.2. Model

We consider a thin film made of $N_{z}$ planes of triangular lattice of L×L sites, stacked in the *z* direction.

We use the following Hamiltonian

(48) $$H=-\sum_{\langle i,j\rangle }J_{i,j}S_{i}\cdot S_{j}-\sum_{<i,j>}I_{i,j}S_{i}^{z}S_{j}^{z}$$

where $S_{i}$ is the Heisenberg spin at the lattice site *i*, $\sum_{\langle i,j\rangle }$ indicates the sum over the nearest-neighbor spin pairs $S_{i}$ and $S_{j}$. The last term, which will be supposed very small, is needed to have a phase transition at a finite temperature for the film with a very small thickness when all exchange interactions $J_{i,j}$ are ferromagnetic.

We suppose that the nn interactions on the surface are $J_{s}$ and $I_{s}$, and all other interactions are ferromagnetic and equal to *J* and *I*. The two surfaces of the film are frustrated if $J_{s}$ is antiferromagnetic ($J_{s}<0$), due to the triangular lattice structure.

### 5.3. Ground State

We suppose here that the spins are classical Heisenberg spins. The classical GS can be calculated as shown below. We recall that for antiferromagnetic systems of quantum spins, the quantum GS though not far from the classical one, cannot be exactly determined because of the quantum fluctuations [6].

For $J_{s}>0$, the GS is ferromagnetic. When $J_{s}$ is antiferromagnetic, the surface when detached from the bulk has the 120-degree ordering and the interior layers have the ferromagnetic ordering. The interaction between the surface spins and those of the beneath layer causes a competition between the collinear configuration and the 120-degree one.

We first determine the ground-state configuration for $I=I_{s}=0.1$ by minimizing the energy of each spin starting from a random spin configuration. This is done by iteration until the convergence is reached. The reader is referred to Ref. [65] for the numerical procedure. In doing so, we obtain the ground-state configuration, without metastable states for the present model.

**Figure 19:**
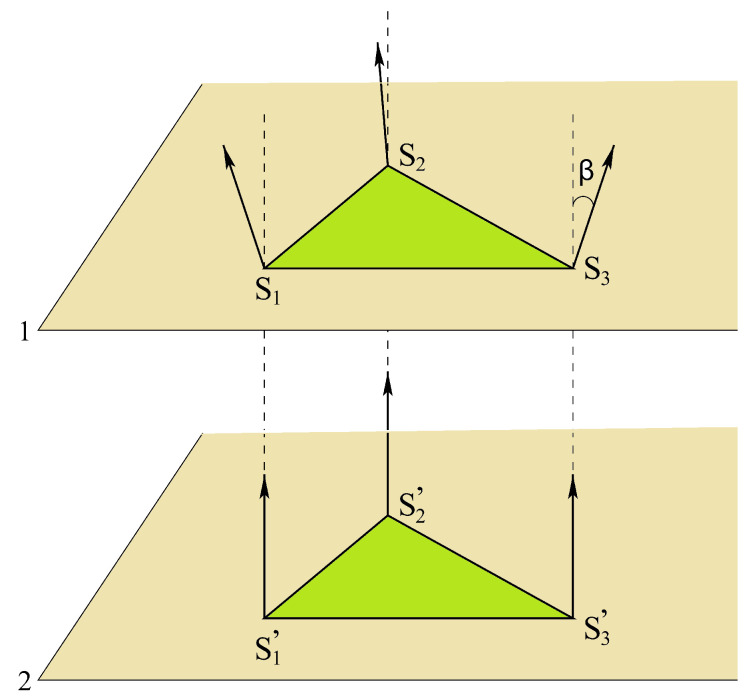
Surface spin configuration: angle between nn spins on layer 1 is equal to α, angle between vertical nn spins is β.

The result shows that when $J_{s}$ is smaller than a critical value $J_{s}^{c}$ the magnetic GS is an “umbrella” form with an angle α between nn surface spins and an angle β between a surface spin and its beneath neighbor (see Figure 19). This structure is due to the interaction of the spins on the beneath layer on the surface spins, acting like an external applied field in the *z* direction. It is obvious that when $|J_{s}|$ is smaller than $|J_{s}^{c}|$ the collinear ferromagnetic GS results in: the frustration is not strong enough to resist the ferromagnetic interaction from the beneath layer.

**Figure 20:**
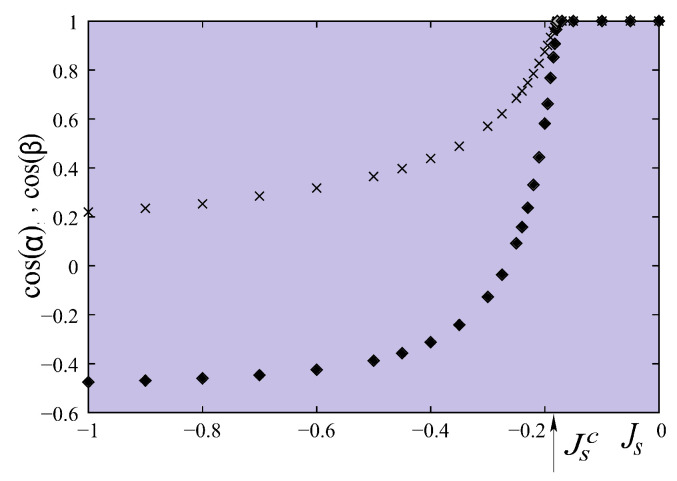
Angles versus surface interaction $J_{s}$: cos(α) (diamonds) and cos(β) (crosses). $J_{s}^{c}$ is indicated by the arrow.

Let us show cos(α) and cos(β) versus $J_{s}$ in Figure 20. The critical value $J_{s}^{c}$ is found between −0.18 and −0.19. This value can be calculated analytically as shown below, by assuming the “umbrella structure”. For the ground-state analysis, we consider just a single cell shown in Figure 19. This is justified by the numerical determination presented above.

We consider the Hamiltonian given by (48). We take that $(J_{i,j}=J_{s},I_{i,j}=I_{s}$) for nn surface spins and all other $\left(J_{i,j}=J>0,I_{i,j}=I>0\right)$ for the inside nn spins including interaction between a surface spin and a nn spin on the second layer.

We number as in Figure 19 $S_{1}$, $S_{2}$ and $S_{3}$ are on the surface layer, $S_{1}^{\prime }$, $S_{2}^{\prime }$ and $S_{3}^{\prime }$ on the second layer. The Hamiltonian for the cell reads

(49) $$\begin{array}{ccc} H_{p} & = & -6\left[J_{s}\left(S_{1}\cdot S_{2}+S_{2}\cdot S_{3}+S_{3}\cdot S_{1}\right)\right.\\ & & +I_{s}\left(S_{1}^{z}S_{2}^{z}+S_{2}^{z}S_{3}^{z}+S_{3}^{z}S_{1}^{z}\right)\\ & + & J\left(S_{1}^{\prime }\cdot S_{2}^{\prime }+S_{2}^{\prime }\cdot S_{3}^{\prime }+S_{3}^{\prime }\cdot S_{1}^{\prime }\right)\\ & & +I\left.\left(S_{1}^{\prime z}S_{2}^{\prime z}+S_{2}^{\prime z}S_{3}^{\prime z}+S_{3}^{\prime z}S_{1}^{\prime z}\right)\right]\\ & - & 2J\left(S_{1}\cdot S_{1}^{\prime }+S_{2}\cdot S_{2}^{\prime }+S_{3}\cdot S_{3}^{\prime }\right)\\ & & -2I\left(S_{1}^{z}S_{1}^{\prime z}+S_{2}^{\prime z}S_{2}^{\prime z}+S_{3}^{z}S_{3}^{\prime z}\right), \end{array}$$

We next decompose each spin into an xy component, which is a vector, and a *z* component $S_{i}=\left(S_{i}^{∥},S_{i}^{z}\right)$. We see that only surface spins have xy vector components. The angle between these xy components of nearest-neighbor surface spins is $\gamma_{i,j}$ which is the projection of α defined above on the xy plane. We have by symmetry

(50) $$\gamma_{1,2}=0,\;\gamma_{2,3}=\frac{2\pi }{3},\;\gamma_{3,1}=\frac{4\pi }{3}.$$

The angles of the spin $S_{i}$ and $S_{i}^{\prime }$ with the *z* axis are by symmetry

$$\left\{\begin{array}{c} \beta_{1}=\beta_{2}=\beta_{3}=\beta ,\\ \beta_{1}^{\prime }=\beta_{2}^{\prime }=\beta_{3}^{\prime }=0, \end{array}\right.$$

The total energy of the cell (49), with $S_{i}=S_{i}^{\prime }=\frac{1}{2}$, can be rewritten as

(51) $$\begin{array}{ccc} H_{p} & = & -\frac{9(J+I)}{2}-\frac{3(J+I)}{2}\cos\beta -\frac{9(J_{s}+I_{s})}{2}\cos^{2}\beta \\ & + & \frac{9J_{s}}{4}\sin^{2}\beta . \end{array}$$

We minimize the cell energy

(52) $$\frac{\partial H_{p}}{\partial \beta }=\left(\frac{27}{2}J_{s}+9I_{s}\right)\cos\beta \sin\beta +\frac{3}{2}\left(J+I\right)\sin\beta \;=\;0$$

which gives the following solution

(53) $$\cos\beta =-\frac{J+I}{9J_{s}+6I_{s}}.$$

For given values of $I_{s}$ and *I*, we see that the solution (53) exists if |cosβ|≤1, namely $J_{s}\leq J_{s}^{c}$ where $J_{s}^{c}$ is the critical value. For $I=-I_{s}=0.1$, one has $J_{s}^{c}\approx -0.1889J$ in perfect agreement with the numerical minimization shown in Figure 20.

The classical GS determined here will be used as input for the ground-state configuration in the case of quantum spins presented below using the Green’s function method.

### 5.4. Results from the Green’s Function Method

We suppose the spins are quantum in this subsection. The details of the formulation for non-collinear spin configurations have been given in Ref. [65]. We just show the results on the surface phase transition and compare with the Monte Carlo results performed on the equivalent classical model.

#### 5.4.1. Phase Transition and Phase Diagram of the Quantum Case

**Figure 21:**
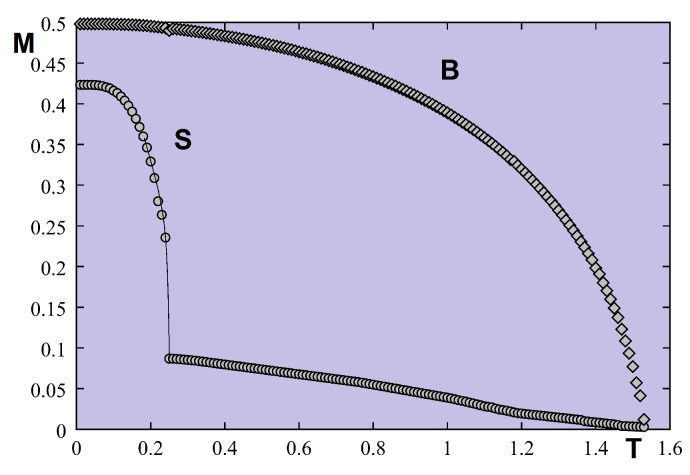
Surface (*S*) and 2nd-layer (*B*) magnetizations versus *T* for the frustrated case where $J_{s}=-0.5$ with $I=-I_{s}=0.1$. See text for comments.

Let us take J=1 as the unit of energy. The temperature is in unit of $J/k_{B}$. We show in Figure 21 the results of the very frustrated case where $J_{s}=-0.5J$ much smaller than $J_{s}^{c}=-0.1889J$. Some remarks are in order: (i) there is a strong spin contraction at T=0 [6] for the surface layer which comes from the antiferromagnetic nature of the in-plane surface interaction $J_{s}$; (ii) the surface magnetization is much smaller than the second-layer one, the surface becomes disordered at a temperature $T_{1}≃0.2557$ while the second layer remains ordered up to $T_{2}≃1.522$.

It is interesting to note that the system is partially disordered for temperatures between $T_{1}$ and $T_{2}$. This result confirms again the existence of the partial disorder in quantum spin systems observed in the bulk [54,69]. Please note that between $T_{1}$ and $T_{2}$, the ordering of the second layer acts as an external field on the first layer, inducing therefore a small value of the surface magnetization.

**Figure 22:**
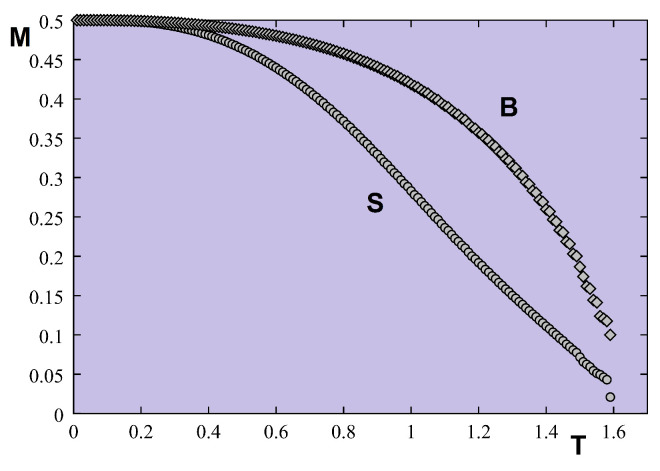
Surface (*S*) and 2nd-layer (*B*) magnetizations versus *T* for the non-frustrated case where $J_{s}=0.5$ with $I=I_{s}=0.1$.

We show now the case of non-frustrated surface in Figure 22 where $J_{s}=0.5$, with $I=I_{s}=0.1$. Though the surface magnetization is smaller than the second-layer magnetization, the result suggests there is only a single transition temperature.

**Figure 23:**
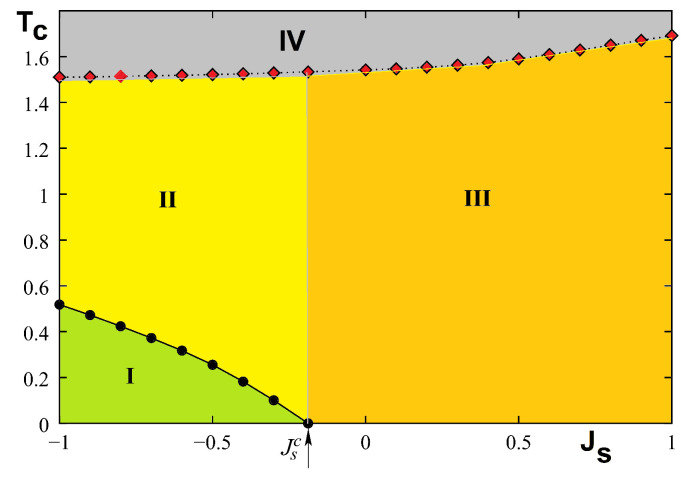
Phase diagram in the space ($J_{s},T$) for the quantum Heisenberg model with $N_{z}=4$, $I=|I_{s}|=0.1$. Phase I is the surface non-collinear phase, phase II has surface disorder and bulk order, phase III and IV are ferromagnetic and paramagnetic phases, respectively.

The phase diagram in the space $\left(J_{s},T\right)$ is shown in Figure 23 where phase I denotes the surface and bulk ordered phase with non collinear spin configuration at the surface. Phase II is the phase where the surface is disordered but the bulk is still ordered, phase III is ferromagnetic, and phase IV is paramagnetic. Please note that the surface transition does not exist for $J_{s}\geq J_{s}^{c}$.

#### 5.4.2. Monte Carlo Results

To study the phase transition occurring at a high temperature, one can use the classical spins and Monte Carlo simulations to obtain the phase diagram for comparison. This is justified since quantum fluctuations are not important at high *T*.

For Monte Carlo simulations (see methods in Refs. [2,70,71,72,73,74]), we use the same Hamiltonian (48) but with the classical Heisenberg spin model of magnitude S=1. We use the film size $L\times L\times N_{z}$ where $N_{z}=4$ is the number of layers, and L=24,36,48,60 to detect the finite-size effects. To reduce the lateral size effect, periodic boundary conditions are employed in the xy planes. The thermodynamic equilibration is done with $10^{6}$ Monte Carlo steps per spin and the averaging time is taken over $2\times 10^{6}$ Monte Carlo steps per spin. J=1 is taken as unit of energy in the following.

**Figure 24:**
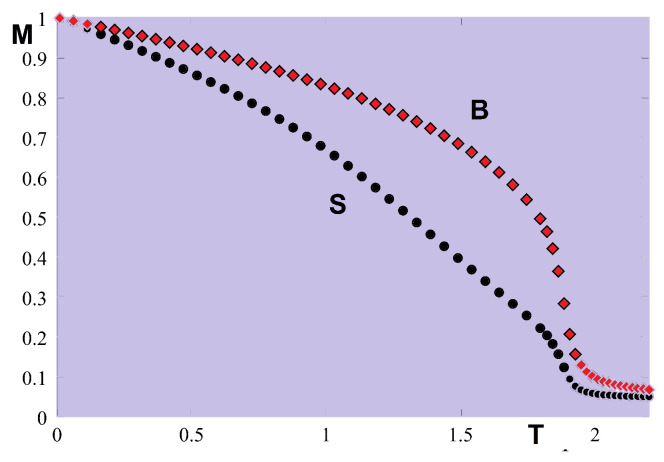
Surface magnetization S (circles) and 2nd-layer magnetization B (diamonds) versus *T* in unit of $J/k_{B}$ for the non-frustrated case $J_{s}=0.5$ with $I=I_{s}=0.1$, L=36.

Figure 24 shows the first- and second-layer magnetizations versus *T* where $J_{s}=0.5$ (no frustration). In this case, there is clearly just a single transition for surface and bulk, as in the quantum case.

**Figure 25:**
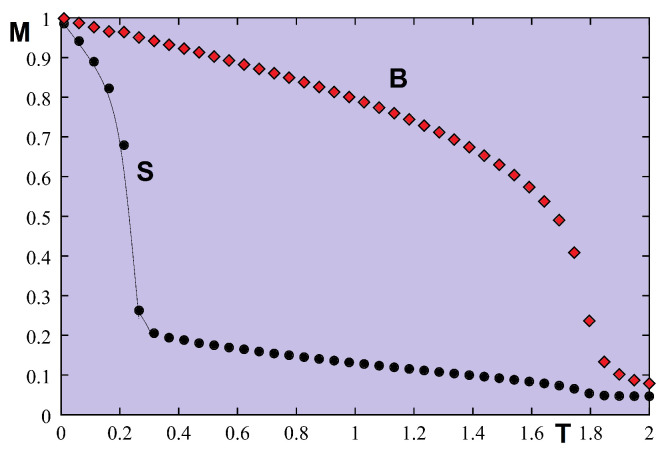
Surface magnetization S (circles) and 2nd-layer magnetization B (diamonds) versus *T* in unit of $J/k_{B}$ for the frustrated case $J_{s}=-0.5$ with $I=-I_{s}=0.1$, L=36.

Let us show in Figure 25 the result of a frustrated case where $J_{s}=-0.5$. As in the quantum case, the surface becomes disordered at a temperature much lower than that for the interior layer.

**Figure 26:**
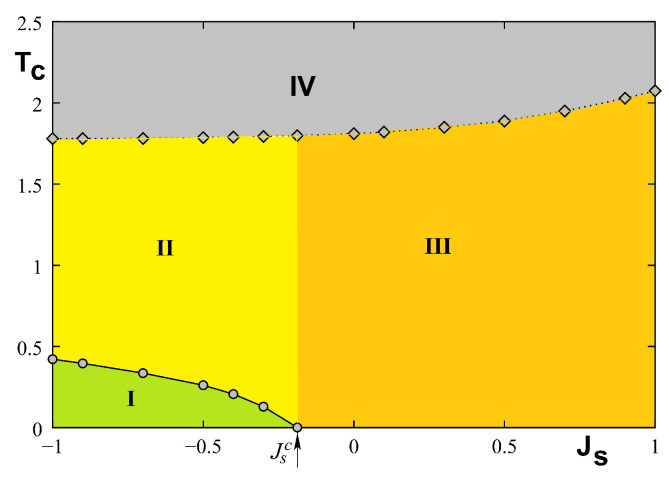
Monte Carlo results of the phase diagram in the space ($J_{s},T$) for the classical Heisenberg model with $N_{z}=4$, $I=|I_{s}|=0.1$. Phases I to IV are defined in Figure 23.

The phase diagram is shown in Figure 26 in the space $\left(J_{s},T\right)$. We see that there is a remarkable similarity to that obtained for the quantum spin model shown in Figure 23.

### 5.5. Frustrated Thin Films

We have also studied frustration effects in an antiferromagnetic fcc Heisenberg film [66]. In this case, the whole film is frustrated due to the geometry of the lattice.

We consider the quantum Heisenberg spins occupying the lattice sites of a film of fcc structure with (001) surfaces. The Hamiltonian reads

(54) $$H=-\sum_{\langle i,j\rangle }J_{i,j}S_{i}\cdot S_{j}-\sum_{i}D_{i}{\left(S_{i}^{z}\right)}^{2}$$

where $S_{i}$ is the spin at the lattice site *i*, the first sum runs over the nn spin pairs $S_{i}$ and $S_{j}$, while the second one runs over all sites. The second terms in the Hamiltonian are Ising-like uniaxial anisotropy terms added to avoid the absence of long-range order of isotropic non-Ising spin model at finite *T* when the film thickness tends to 1 [64].

Hereafter, let $J_{s}$ be the interaction between two nn surface spins, J=−1 (antiferromagnetic) for all other interactions.

**Figure 27:**
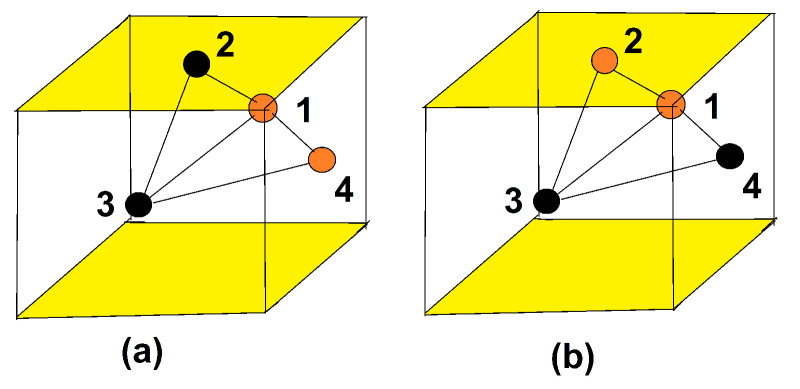
Ground-state spin configuration of the surface fcc cell: (**a**) type I for $J_{s}<-0.5$; (**b**) type II for $J_{s}>-0.5$ (J=−1). Black and red circles indicate up and down spins, respectively. The vertical axis is the *z* axis. The *x* and *y* axes along the cube in the yellow plane form with the *z* axis a direct triad.

The GS depends $J_{s}$ with a critical value $J_{s}^{c}=-0.5$ at which the ordering of type I coexists with ordering of type II (see Figure 27). The demonstration has been given in Ref. [66].

For $J_{s}<J_{s}^{c}$, the spins in each yz plane are parallel while spins in adjacent yz planes are antiparallel (Figure 27a). This ordering will be called hereafter “ordering of type I”: in the *x* direction the ferromagnetic planes are antiferromagnetically coupled as shown in this figure. Of course, there is a degenerate configuration where the ferromagnetic planes are antiferromagnetically ordered in the *y* direction. Please note that the surface layer has an antiferromagnetic ordering for both configurations. The degeneracy of type I is therefore 4 including the reversal of all spins.

For $J_{s}>J_{s}^{c}$, the spins in each xy plane is ferromagnetic. The adjacent xy planes have an antiferromagnetic ordering in the *z* direction perpendicular to the film surface. This will be called hereafter “ordering of type II”. Please note that the surface layer is then ferromagnetic (Figure 27b). The degeneracy of type II is 2 due to the reversal of all spins.

**Figure 28:**
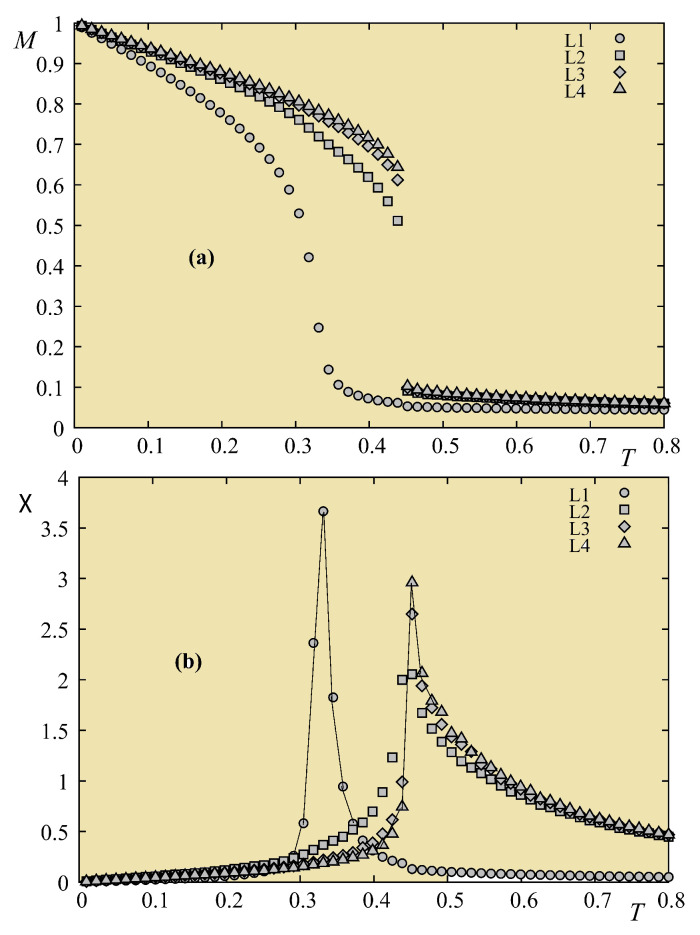
(**a**) Layer sublattice magnetizations and (**b**) layer sublattice susceptibilities of first two cells vs. *T* for $J_{s}=-0.5$, D=0.1 (J=−1). $L_{1}$ to $L_{4}$ indicate data for layer 1 to 4. The susceptibility of layer 1 is divided by a factor 5 for presentation scale convenience.

**Figure 29:**
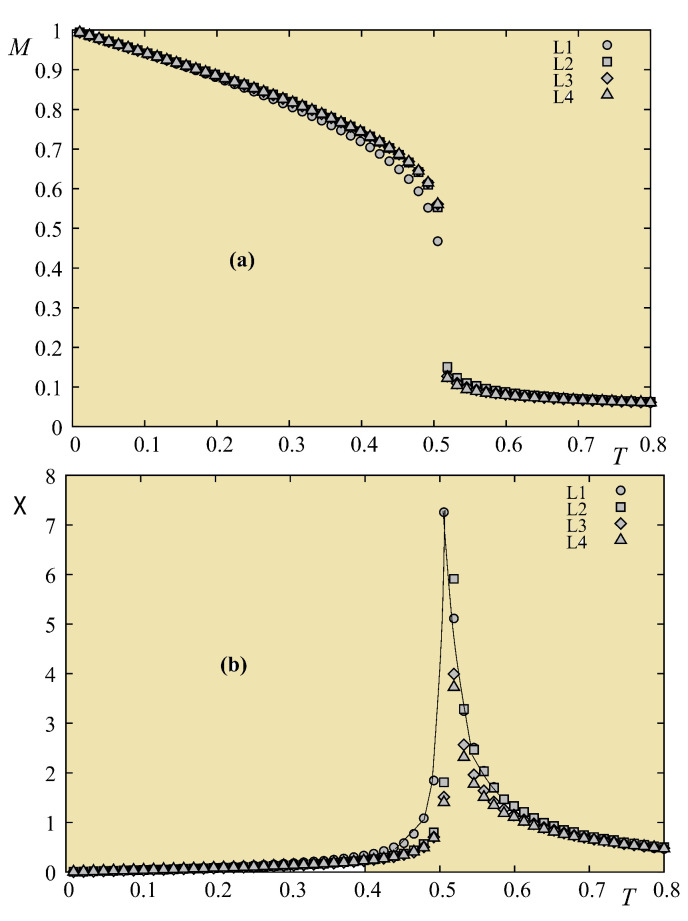
(**a**) Layer sublattice magnetizations and (**b**) layer sublattice susceptibilities of first two cells vs. *T* for $J_{s}=-0.8$ with D=0.1 (J=−1). $L_{1}$ to $L_{4}$ indicate data for layer 1 to 4.

**Figure 30:**
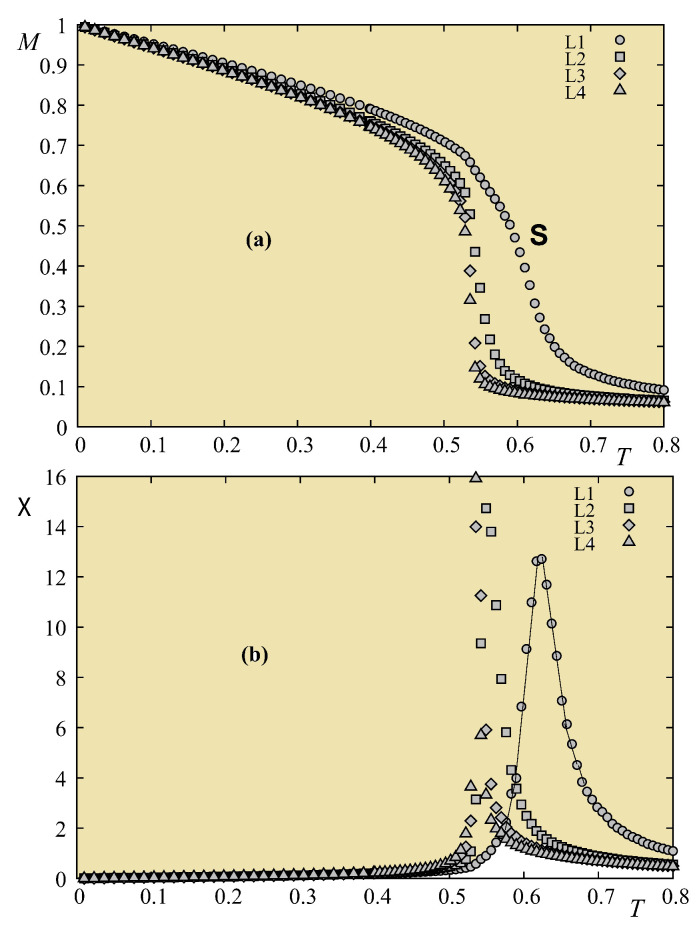
(**a**) Layer sublattice magnetizations and (**b**) layer sublattice susceptibilities of first two cells vs. *T* for $J_{s}=-1$ with D=0.1 (J=−1). $L_{1}$ to $L_{4}$ indicate data for layer 1 to 4.

Monte Carlo simulations have been used to study the phase transition in this frustrated film. We just show below three typical cases, at and far from $J_{s}^{c}$. Figure 28 shows the sublattice layer magnetizations at $J_{s}^{c}=-0.5$ where one sees that the surface layer undergoes a transition at a temperature lower than the interior ones. Far from this value there is a single phase transition as seen in Figure 29. However, when $J_{s}$ is negatively stronger, we have a hard surface, namely the surface undergoes a phase transition at a *T* higher than that for the interior layer transition. This is seen in Figure 30.

**Figure 31:**
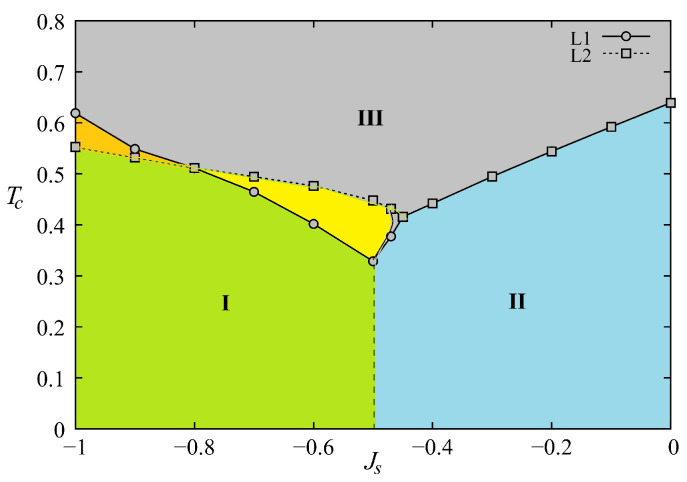
Phase diagram in the space $\left(J_{s},T_{c}\right)$ with D=0.1. I and II denote ordering of type I and II defined in in Figure 27. III is paramagnetic phase. The yellow (gold) zone indicates the surface (bulk) disordering while bulk (surface) is still ordered. The discontinued vertical line is a first-order line. See text for comments.

The phase diagram is shown in Figure 31.

Please note that near the phase boundary $J_{s}^{c}$ ($-0.5\leq J_{s}\leq -0.43$) a reentrant phase is found between phases I and II (not seen with the figure scale). As said in the 2D exactly solved models above, one must be careful while examining the very small region near the phase boundary $J_{s}^{c}$ where unexpected phenomena can occur. This is the case here.

We have studied the nature of the phase transition by using the Monte Carlo multi-histogram technique [72,73,74]. Critical exponents are found to have values between 2D and 3D universality classes. The reader is referred to Ref. [66] for details. The criticality of thin films is treated in Section 6 below.

### 5.6. Helimagnetic Films

Bulk helimagnets have been studied a long time ago [75,76,77]. A simple helimagnetic order resulting from the competition between the nn and nnn interactions is shown in Section 2.2. Helimagnetic films are seen therefore as frustrated films.

We have recently used the Green’s function method and Monte Carlo simulations to study helimagnetic films in zero field [63,78] and in a perpendicular field [79]. We summarize here some results and emphasize their importance.

Consider the following helimagnetic Hamiltonian

(55) $$H=-\sum_{\langle i,j\rangle }J_{i,j}S_{i}\cdot S_{j}-\sum_{i}H\cdot S_{i}$$

where $J_{i,j}$ is the interaction between two spins $S_{i}$ and $S_{j}$ occupying the lattice sites *i* and *j* and H denotes an external magnetic field applied along the *c* axis. We suppose the ferromagnetic interaction $J_{1}$ between nn everywhere. To generate a helical configuration in the *c* direction, one must take into account an antiferromagnetic interaction $J_{2}$ between nnn in the *c* direction, in addition to $J_{1}$. Hereafter, we suppose $J_{2}$ is the same at the surface and in the bulk for simplicity. Please note that in the bulk in zero field, the helical angle along the *c* axis is given by $\cos\alpha =-\frac{J_{1}}{4J_{2}}$ [see Equation (6)] for a simple cubic lattice when $|J_{2}|>0.25J_{1}$ ($J_{2}<0)$. Below this value, the ferromagnetic phase is stable.

**Figure 32:**
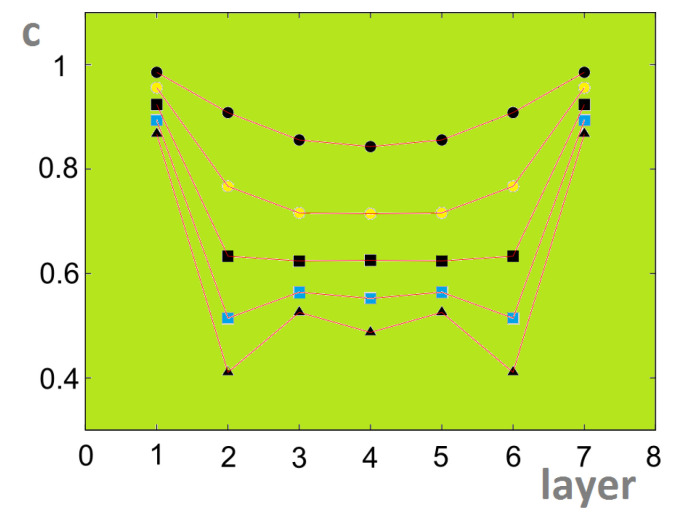
Non-uniform angles across the film: *C* represents cosines of $\alpha_{1}=\theta_{1}-\theta_{2}$, …, $\alpha_{7}=\theta_{7}-\theta_{8}$ for $J_{2}/J_{1}=-1.2,-1.4,-1.6,-1.8,-2$ (from top) with $N_{z}=8$.

In zero field the helical angle in a thin film has been shown [63] to be strongly modified near the surface as presented in Figure 32.

**Figure 33:**
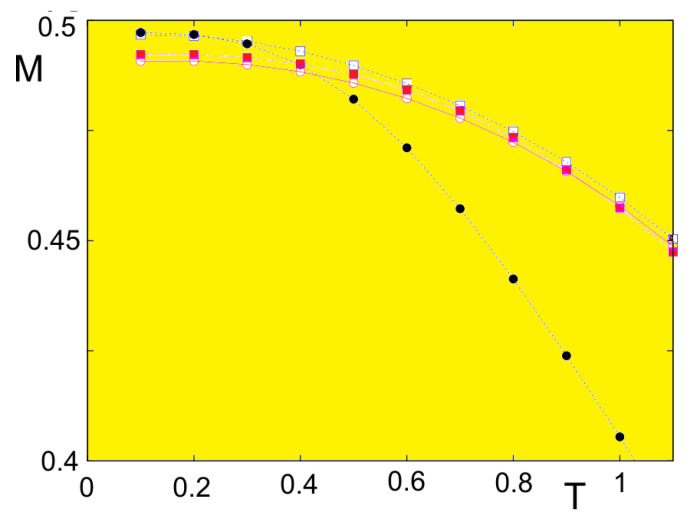
Layer magnetizations versus *T* for $J_{2}/J_{1}=-1.4$ with d=0.1, $N_{z}=8$. Black circles, blue void squares, magenta squares, and red void circles are for first, second, third, and fourth layers, respectively. Magnetization crossovers at low *T* are seen. See text for explanation.

Some results from the laborious Green’s function are shown in Figure 33. To have a long-range ordering at finite *T*, we added an anisotropic term $d\;S_{i}^{z}\;S_{j}^{z}$ in the Hamiltonian where $d<<J_{1}$. We observe in Figure 33 the crossover of the layer magnetizations at low *T*. This is due to quantum fluctuations which are different for each layer, depending on the antiferromagnetic interaction strength (namely the so-called zero-point spin contractions, see Ref. [6]). Without such a theoretical insight, it would be difficult to understand experimental data when one observes this phenomenon at low *T*.

In an applied field [79], we have observed a new phenomenon, namely a partial phase transition in the helimagnetic film. Contrary to what has been shown above (surface phase transition below or above the bulk one), here we have each single interior layer undergoes a separate transition. Theoretically, we can understand this phenomenon by the following fact: under an applied magnetic field, due to the surface effect shown in Figure 32 the spins of each layer in the GS make an angle with the *c* axis different from those of the other layers of the film (in fact we examine only layers of half of the film, the other half is symmetric because of the symmetry of the two surfaces). When the temperature increases, the layers with large xy spin-components undergo a phase transition where the transverse (in-plane) xy ordering is destroyed. This “in-plane” transition can occur at a temperature because the xy spin-components do not depend on the field. Other layers with small xy spin-components, not large enough to have an xy ordering, do not make a transition. In addition, these layers have large *z* components, they cannot undergo a transition because the ordering in $S^{z}$ is maintained by the applied field.

The transition of several layers with the destruction of the xy ordering, not all layers, is a new phenomenon found in this work with our helimagnetic films in a perpendicular field. Real helimagnetic materials often have interactions more complicated than those in the model studied here, but the important ingredient is the non-uniformity of the spin configuration in an applied field, whatever the interactions are. The clear physical pictures given in our present analysis are believed to be useful in the search for the interpretation of experimental data.

## 6. Criticality of Thin Films

One of the important fundamental questions in surface physics is the criticality of the phase transition in thin films.

To clarify this aspect, we studied the critical behavior of magnetic thin films with varying film thickness [80]. In that work, we have studied the ferromagnetic Ising model with the high-resolution multiple histogram Monte Carlo method [72,73,74]. We found that though the 2D behavior remains dominant at small thicknesses, there is a systematic continuous deviation of the critical exponents from their 2D values. We explained these deviations using the concept of “effective” exponents proposed by Capehart and Fisher [81] in a finite-size analysis. The variation the critical temperature with the film thickness obtained by our Monte Carlo simulations is in excellent agreement with their prediction.

Let us summarize here this work.

We consider a film made from a ferromagnetic simple cubic lattice of size $L\times L\times N_{z}$. Periodic boundary conditions (PBC) are used in the xy planes to reduce the lateral size effect. The *z* direction is limited by the film thickness $N_{z}$. $N_{z}=1$ corresponds to a 2D square lattice.

The Hamiltonian is written as

(56) $$H=-\sum_{\langle i,j\rangle }J_{i,j}\sigma_{i}\cdot \sigma_{j}$$

where $\sigma_{i}=\pm 1$ is the Ising spin at the lattice site *i*, and the sum is performed over the nn spin pairs $\sigma_{i}$ and $\sigma_{j}$. $J_{i,j}=J=1$ for all spin pairs.

Using the high-precision multi-histogram Monte Carlo technique [72,73,74] we have calculated various critical exponents as functions of the film thickness using the finite-size scaling [82] described as follows. In Monte Carlo simulations, one calculates by the multiple histogram technique averaged total energy 〈E〉, specific heat $C_{v}$, averaged order parameter 〈M〉 (*M*: magnetization of the system), susceptibility χ, first-order cumulant of the energy $C_{U}$, and $n^{th}$-order cumulant $V_{n}$ of the order parameter, for n=1 and 2. These quantities are defined as

(57) $$\begin{array}{ccc} \langle E\rangle & = & \langle H\rangle , \end{array}$$

(58) $$\begin{array}{ccc} C_{v} & = & \frac{1}{k_{B}T^{2}}\left(\langle E^{2}\rangle -{\langle E\rangle }^{2}\right), \end{array}$$

(59) $$\begin{array}{ccc} M & = & \frac{1}{L\times L\times N_{T}}\langle \sum_{i}\sigma_{i}\rangle , \end{array}$$

(60) $$\begin{array}{ccc} \chi & = & \frac{1}{k_{B}T}\left(\langle M^{2}\rangle -{\langle M\rangle }^{2}\right), \end{array}$$

(61) $$\begin{array}{ccc} C_{U} & = & 1-\frac{\langle E^{4}\rangle }{3{\langle E^{2}\rangle }^{2}}, \end{array}$$

(62) $$\begin{array}{ccc} V_{n} & = & \frac{\partial \ln M^{n}}{\partial (1/k_{B}T)}=\langle E\rangle -\frac{\langle M^{n}E\rangle }{\langle M^{n}\rangle }. \end{array}$$

where 〈…〉 indicates the thermal average at a given *T*.

Let us summarize the multi-histogram technique [72,73,74]. With this technique, we calculate the probability at a temperature $T_{0}$ using the energy histogram recorded during the simulation. The probability at temperatures around $T_{0}$ can be deduced. For the multi-histogram technique, we should make many simulations with different $T_{0}$. The combination of these results gives a good probability as a continuous function of temperature. Thermal averages of physical quantities are calculated as continuous functions of *T*, and the results are valid over a much wider range of temperature than the results from the single histogram technique.

Let $H_{j}\left(E\right)$ be the energy histogram recorded during the *j*-th simulation. The overall probability distribution [74] at temperature *T* obtained from *n* independent simulations, each with $N_{j}$ configurations, is given by

(63) $$P\left(E,T\right)=\frac{\sum_{j=1}^{n}H_{j}\left(E\right)\exp\left[E/k_{B}T\right]}{\sum_{j=1}^{n}N_{j}\exp\left[E/k_{B}T_{j}-f_{j}\right]},$$

where

(64) $$\exp\left[f_{i}\right]=\sum_{E}P\left(E,T_{i}\right).$$

The thermal average of a physical quantity *A* is then calculated by

(65) $$\langle A\left(T\right)\rangle =\sum_{E}A\;P\left(E,T\right)/z\left(T\right),$$

in which

(66) $$z\left(T\right)=\sum_{E}P\left(E,T\right).$$

In our simulations, the xy lattice sizes L×L with *L* = 20, 24, 30, …, 80 have been used. For $N_{z}=3$, sizes up to 160×160 have been used to evaluate corrections to scaling. In each simulation, the standard Metropolis MC simulations are used first to localize for each size the transition temperatures for specific heat and for susceptibility. The equilibrating time is from 200,000 to 400,000 MC steps/spin and the averaging time is from 500,000 to 1,000,000 MC steps/spin. We record next histograms at 8 different temperatures $T_{j}\left(L\right)$ around the transition temperatures with 2 million MC steps/spin, after equilibrating the system with 1 million MC steps/spin. Finally, we record again histograms at 8 different temperatures around the new transition temperatures with $2\times 10^{6}$ and $4\times 10^{6}$ MC steps/spin for equilibrating and averaging time, respectively. Such an iteration procedure gives extremely good results for systems studied so far. Errors shown in the following have been estimated using statistical errors, which are very small thanks to our multiple histogram procedure, and fitting errors given by fitting software.

Let us discuss first the 3D case where all dimensions can go to infinity. Consider a system of size $L^{d}$ where *d* is the space dimension. In the simulation for a finite *L*, one can identify the pseudo “transition” temperatures by the maxima of $C_{v}$ and χ, …. These maxima in general take place at close, but not the same, temperatures. When *L* tends to infinity, these pseudo “transition” temperatures tend to the “real” transition temperature $T_{c}\left(\infty \right)$. Thus, when we examine the maxima of $V_{n}$, $C_{v}$ and χ, we are not at $T_{c}\left(\infty \right)$. We have to bear this in mind for the discussion given in the following. Now, let us define the reduced temperature, which is the “distance” from $T_{c}\left(\infty \right)$, by

(67) $$t=\frac{T-T_{c}\left(\infty \right)}{T_{c}\left(\infty \right)}$$

In the finite-size scaling theory, the following scaling relations are valid for large values of *L* at their respective ’transition’ temperatures $T_{c}\left(L\right)$ (see details in Ref. [83]):

(68) $$V_{1}^{\max}\propto L^{1/\nu },\;V_{2}^{\max}\propto L^{1/\nu },$$

(69) $$C_{v}^{\max}=C_{0}+C_{1}L^{\alpha /\nu }$$

(70) $$\chi^{\max}\propto L^{\gamma /\nu }$$

(71) $$C_{U}=C_{U}\left[T_{c}\left(\infty \right)\right]+AL^{-\alpha /\nu },$$

(72) $$M_{T_{c}\left(\infty \right)}\propto L^{-\beta /\nu }$$

(73) $$T_{c}\left(L\right)=T_{c}\left(\infty \right)+C_{A}L^{-1/\nu },$$

where *A*, $C_{0}$, $C_{1}$ and $C_{A}$ are constants. The exponent ν is calculated independently from $V_{1}^{\max}$ and $V_{2}^{\max}$. Using this value we calculate γ from the scaling of $\chi^{\max}$, and α from $C_{v}^{\max}$. The value of $T_{c}\left(\infty \right)$ can be calculated using the last expression. Next, with $T_{c}\left(\infty \right)$ we can calculate β from $M_{T_{c}\left(\infty \right)}$. We emphasize that the Rushbrooke scaling law α+2β+γ=2 is in principle verified [82]. This is a way to verify the obtained critical exponents.

**Figure 34:**
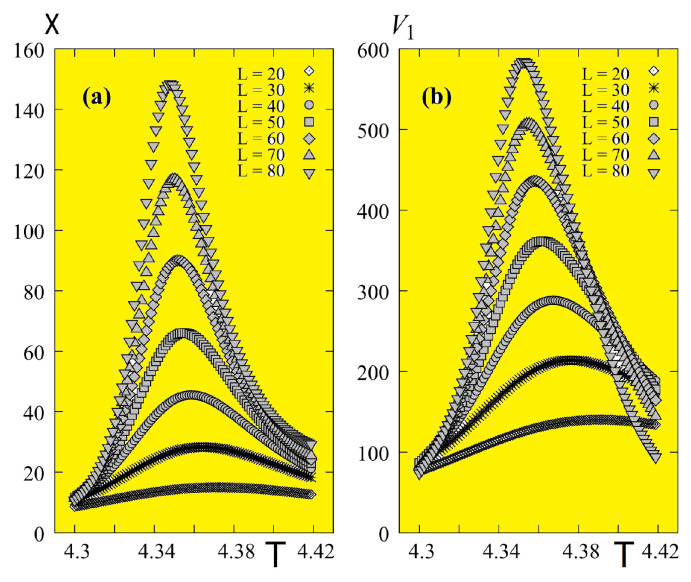
(**a**) Susceptibility and (**b**) $V_{1}$, as functions of *T* for several *L* with $N_{z}=11$, obtained by multiple histogram technique.

Results obtained from multiple histograms described above are shown in Figure 34 for the susceptibility and the first derivative $V_{1}$ calculated with the continuous *T*, using Equations (63)–(66), at temperatures around their maxima, with several sizes L×L (L=20−80).

**Figure 35:**
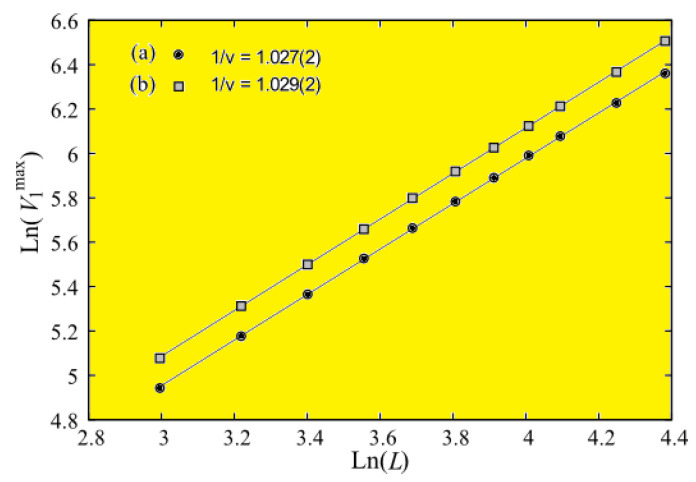
$\ln V_{1}^{\max}$ versus *L* in the ln−ln scale for $N_{z}=11$ (**a**) without PBC in *z* direction (**b**) with PBC in *z* direction. The slope values are given on the plot.

The calculation of ν is shown in Figure 35 for $N_{z}=11$ to illustrate the precision of the method: the slope of the “perfect” straight line of our data points gives 1/ν.

Other critical exponents are summarized in Table 1. Our results indicate a very small but systematic deviation of the 2D critical exponents with increasing thickness. Note the precision of the 2D case ($N_{z}=1$) with respect to the exact result: we have $T_{c}\left(L=\infty ,N_{z}=1\right)=2.2699\pm 0.0005$. The exact value of $T_{c}\left(\infty \right)$ is 2.26919 by solving the equation $\sinh^{2}\left(2J/T_{c}\right)=1$. The excellent agreement of our result shows no doubt the efficiency of the multiple histogram technique used in our work.

**Figure 36:**
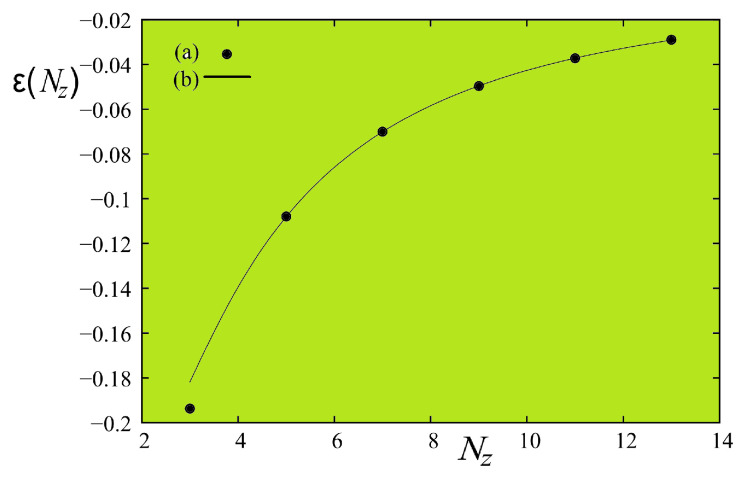
Critical temperature at infinite lateral linear size *L* versus $N_{z}$. Data points are Monte Carlo results, continuous line is from Equation (75).

We show now the theory of Capehart and Fisher [81] on the variation of the critical temperature with $N_{z}$. Defining the critical-point shift due to the finite size by

(74) $$\varepsilon \left(N_{z}\right)=\left[T_{c}\left(L=\infty ,N_{z}\right)-T_{c}\left(3D\right)\right]/T_{c}\left(3D\right)$$

the authors [81] showed that

(75) $$\varepsilon \left(N_{z}\right)\approx \frac{b}{N_{z}^{1/\nu }}\left[1+a/N_{z}\right]$$

where ν=0.6289 (3D value). Using $T_{c}\left(3D\right)=4.51$, we fit the above formula with $T_{c}\left(L=\infty ,N_{z}\right)$ taken from Table 1, we obtain a=−1.37572 and b=−1.92629. The Monte Carlo results and the fitted curve are shown in Figure 36. The prediction of Capehart and Fisher is thus very well verified.

Note finally that the PBC in the *z* direction does not change the result if we do not apply the finite-size scaling in that direction [80].

We have also shown that by decreasing the film thickness, a first-order transition in a frustrated fcc Ising thin film can become a second-order transition [84].

## 7. Skyrmions in Thin Films and Superlattices

Skyrmions are topological excitations in a spin system. They result from the competition between different interactions in an applied magnetic field.

The skyrmion has been named after Skyrme [85,86] for formulating a topological soliton to model a particle in nuclear physics. Various kinds of skyrmions have been shown to exist in condensed matter [87,88,89,90,91,92,93,94]. For a review on the history of chiral skyrmions see the introductory part of Ref. [95].

We consider in this section the case of a sheet of square lattice of size N×N, occupied by Heisenberg spins interacting via a nn ferromagnetic interaction *J* and a nn Dzyaloshinskii-Moriya (DM) interaction [24,25]. The Hamiltonian is written as

(76) $$\begin{array}{ccc} H & = & -J\sum_{\langle ij\rangle }S_{i}\cdot S_{j}+D\hat{z}\cdot \sum_{i}S_{i}\wedge \left(S_{i+x}+S_{i+y}\right)\\ & & -H\sum_{i}S_{i}^{z} \end{array}$$

where the *D* vector of the DM interaction is chosen along the $\hat{z}$ direction perpendicular to the plane.

In zero field we have studied the spin waves and layer magnetizations at T=0 and at finite *T* [96]. The results show that the DM interaction strongly affects the first mode of the SW spectrum. Skyrmions appear only when an external field is applied perpendicular to the film, as seen in the following.

**Figure 37:**
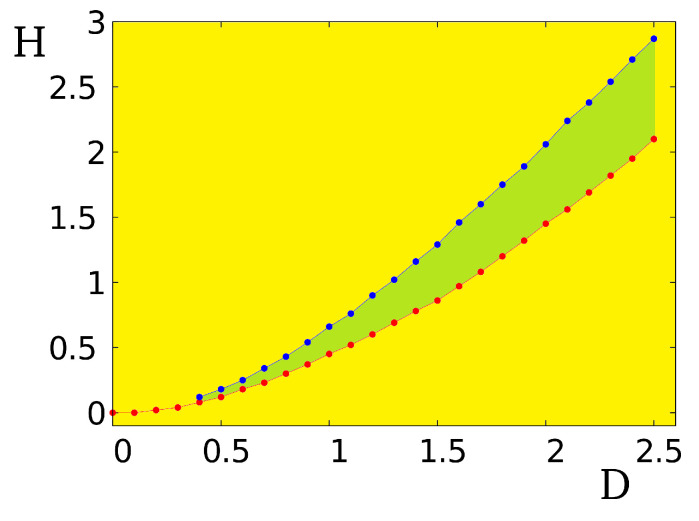
Ground-state phase diagram in the (D,H) plane for lattice size 100×100.

With H≠0, we minimize numerically the above Hamiltonian for a given pair (H,D), taking J=1 as unit, we obtain the GS configuration of the system. The phase diagram is shown in Figure 37. Above the blue line is the field-induced ferromagnetic phase. Below the red line is the labyrinth phase with a mixing of skyrmions and rectangular domains. The skyrmion crystal phase is found in a narrow region between these two lines, down to infinitesimal *D*.

**Figure 38:**
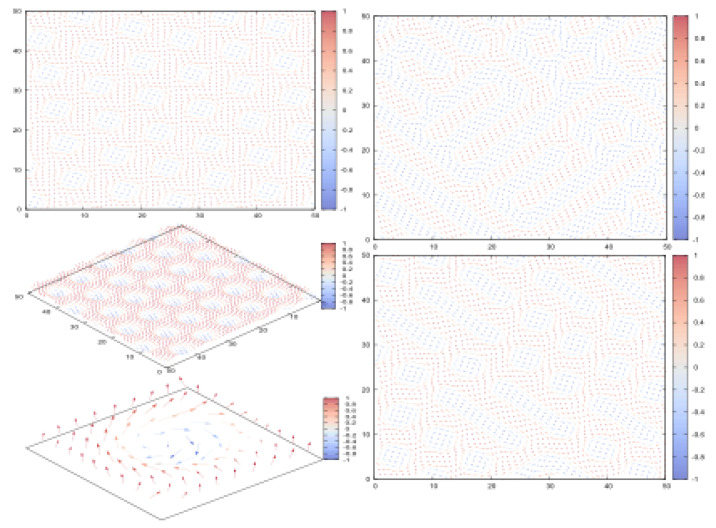
Left: A skyrmion crystal is observed for D/J=1 and H/J=0.5. From above, skyrmion crystal viewed in the xy plane, a 3D view, zoom of the structure of a single vortex. The value of $S_{z}$ is indicated on the color scale. Right top: GS for D=1 and H=0, a mixing of domains of long and round islands. Right bottom: GS for D=1 and H=0.25, a mixing of domains of long islands and vortices. We call these structures the “labyrinth phase”.

Let us show an example of the skyrmion crystal observed at (D=1,H=0.5) (Figure 38 left). We see that the skyrmions form a crystal of triangular lattice. The size of each skyrmion depends on the ratio H/D.

The labyrinth phase below the red line of Figure 37 is shown in Figure 38 (right) for the cases (D=1,H=0.25) and (D=1,H=0).

We wish to study the effect of temperature on the skyrmion crystal. To that end, we define an order parameter of the crystal as follows: we want to see the stability of the skyrmions at a finite *T* so we make the projection of the actual spin configuration at time *t* at temperature *T* on the GS configuration. We should average this projection over many Monte Carlo steps per spin. The order parameter *M* is

(77) $$M\left(T\right)=\frac{1}{N^{2}\left(t_{a}-t_{0}\right)}\sum_{i}\left|\sum_{t=t_{0}}^{t_{a}}S_{i}\left(T,t\right)\cdot S_{i}^{0}\left(T=0\right)\right|$$

where $S_{i}\left(T,t\right)$ is the *i*-th spin at time *t*, at temperature *T*, and $S_{i}\left(T=0\right)$ is its state in the GS. By this definition, we see that the order parameter M(T) is close to 1 at very low *T* where each spin is only weakly deviated from its state in the GS, and M(T) is zero when every spin strongly fluctuates in the paramagnetic state.

**Figure 39:**
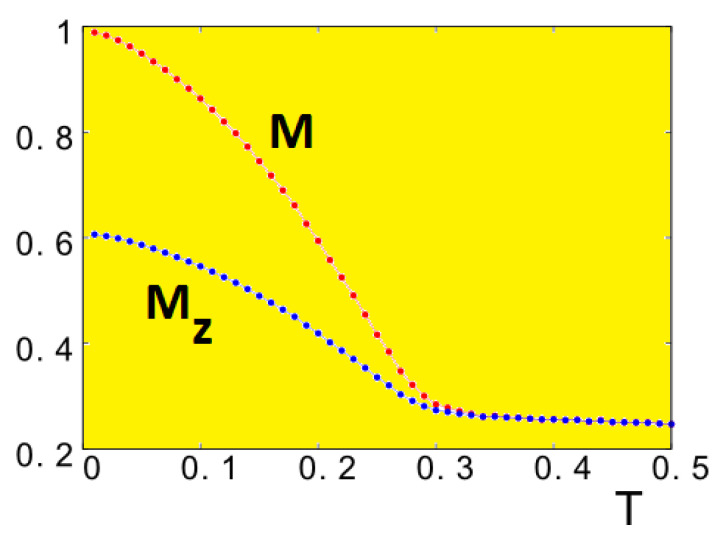
Order parameter *M* (red symbols) defined in Equation (77) versus *T*, for H=0.5 and lattice size 1800×1800. The projection $M_{z}$ versus *T* (blue symbols) of $S_{z}$ on $S_{0}^{z}$ of the GS as defined in Equation (77) but for the *z* components only.

We show in Figure 39 the dependence of *M* and $M_{z}$ on *T* which indicates that the skyrmion crystal remains ordered up to a finite *T*. This stability at finite *T* may be important for transport applications.

We have carried out a finite-size scaling on the phase transition at $T_{c}$. We have observed that from the size 800×800, all curves coincide within statistical errors. Thus, there is no observable finite-size effects for larger lattice sizes.

An important feature of topological systems such as the present system and disordered systems in general (spin glasses, random-field models, …) is the relaxation behavior. In general, they do not follow the simple exponential law [97]. We have studied the relaxation time of the skyrmion crystal and found that it follows a stretched exponential law [98].

The DM interaction has been shown to generate a skyrmion crystal in a 2D lattice. However, skyrmions have been shown to exist in various kinds of lattices [99,100,101,102] and in crystal liquids [87,88,89]. Experimental observations of skyrmion lattices have been realized in MnSi in 2009 [93,94] and in doped semiconductors in 2010 [92]. Also, the existence of skyrmion crystals have been found in thin films [90,91] and direct observation of the skyrmion Hall effect has been realized [103]. In addition, artificial skyrmion lattices have been devised for room temperatures [104].

It is noted that applications of skyrmions in spintronics have been largely discussed and their advantages compared to early magnetic devices such as magnetic bubbles have been pointed out in a recent review by W. Kang et al. [105]. Among the most important applications of skyrmions, let us mention skyrmion-based racetrack memory [106], skyrmion-based logic gates [107,108], skyrmion-based transistor [109,110,111] and skyrmion-based artificial synapse and neuron devices [112,113].

Finally, we mention that we have found skyrmions confined at the interface of a superlattice composed alternately of a ferromagnetic film and a ferroelectric film [114,115,116]. These results may have important applications.

## 8. Conclusions

In this review, we have shown several studied cases on the frustration effects in two dimensions and in magnetic thin films.

The main idea of the review is to show some frustrated magnetic systems which present several common interesting features. These features are discovered by solving exactly some 2D Ising frustrated models, they occur near the frontier of two competing phases of different ground-state orderings. Without frustration, such frontiers do not exist. Among the striking features, one can mention the “partial disorder”, namely several spins stay disordered in coexistence with ordered spins at equilibrium, the “reentrance”, namely a paramagnetic phase exists between two ordered phases in a small region of temperature, and “disorder lines”, namely lines on which the system loses one dimension to allow for a symmetry change from one side to the other. Such beautiful phenomena can only be uncovered and understood by means of exact mathematical solutions.

We have next studied frustrated magnetic systems close to the 2D solvable systems. We have chosen thin magnetic thin films with Ising or other spin models that are not exactly solvable. Guided by the insights of exactly solvable systems, we have introduced ingredients in the Hamiltonian to find some striking phenomena mentioned above: we have seen in thin films partial disorder (surface disorder coexisting with bulk order), reentrance at phase boundaries in fcc antiferromagnetic films. Thin films have their own interest such as surface spin rearrangement (helimagnetic films) and surface effects on their thermodynamic properties. Those points have been reviewed here.

The surface effects have been studied by means of the Green’s function method for frustrated non-collinear spin systems. Monte Carlo simulations have also been used to elucidate many physical phenomena where analytical methods cannot be used. Surface spin waves, surface magnetization, and surface phase transition have been analyzed as functions of interactions, temperature, and applied field.

We have also treated the question of surface criticality. Results of our works show that critical exponents in thin films depend on the film thickness, their values lie between the values of 2D and 3D universality classes. Recent results on skyrmions have also been reviewed in this paper. One of the striking findings is the discovery of a skyrmion crystal in a spin system with DM interaction in competition with an exchange interaction, in a field. This skyrmion crystal is shown to be stable at finite temperature.

To conclude, we would like to say that investigations on the subjects discussed above continue intensively today. Please note that there is an enormous number of investigations of other researchers on the above subjects and on other subjects concerning frustrated magnetic thin films. We have mentioned these works in our original papers, but to keep the paper length reasonable we did not present them here. Also, for the same reason, we have cited only a limited number of experiments and applications in this review.

## Figures and Tables

**Table 1:** Critical exponents, effective dimension, and critical temperature at infinite xy dimension for thickness $N_{z}$ from 1 to 13.

$N_{z}$	ν	γ	α	β	$d_{\mathrm{eff}}$	$T_{c}\left(L=\infty ,N_{z}\right)$
1	0.9990±0.0028	1.7520±0.0062	0.00199±0.00279	0.1266±0.0049	2.0000±0.0028	2.2699±0.0005
3	0.9922±0.0019	1.7377±0.0035	0.00222±0.00192	0.1452±0.0040	2.0135±0.0019	3.6365±0.0024
5	0.9876±0.0023	1.7230±0.0069	0.00222±0.00234	0.1639±0.0051	2.0230±0.0023	4.0234±0.0028
7	0.9828±0.0024	1.7042±0.0087	0.00223±0.00238	0.1798±0.0069	2.0328±0.0024	4.1939±0.0032
9	0.9780±0.0016	1.6736±0.0084	0.00224±0.00161	0.1904±0.0071	2.0426±0.0016	4.2859±0.0022
11	0.9733±0.0025	1.6354±0.0083	0.00224±0.00256	0.1995±0.0088	2.0526±0.0026	4.3418±0.0032
13	0.9692±0.0026	1.6122±0.0102	0.00226±0.00268	0.2059±0.0092	2.0613±0.0027	4.3792±0.0034

## Abbreviations

The following abbreviations are used in this manuscript:

bcc	body-centered cubic (lattice)
DM	Dzyaloshinskii-Moriya
fcc	face-centered cubic (lattice)
GS	ground state
hcp	hexagonal close-packed (lattice)
MC	Monte Carlo
nn	nearest neighbors
nnn	next-nearest neighbors
PBC	periodic boundary conditions
SW	spin wave
2D	two dimensions
3D	three dimensions
